# Reconciling Apparent Conflicts between Mitochondrial and Nuclear Phylogenies in African Elephants

**DOI:** 10.1371/journal.pone.0020642

**Published:** 2011-06-08

**Authors:** Yasuko Ishida, Taras K. Oleksyk, Nicholas J. Georgiadis, Victor A. David, Kai Zhao, Robert M. Stephens, Sergios-Orestis Kolokotronis, Alfred L. Roca

**Affiliations:** 1 Department of Animal Sciences, University of Illinois at Urbana-Champaign, Urbana, Illinois, United States of America; 2 Department of Biology, University of Puerto Rico at Mayagüez, Mayagüez, Puerto Rico; 3 Bole and Klingenstein Foundation, Cody, Wyoming, United States of America; 4 Laboratory of Genomic Diversity, National Cancer Institute at Frederick, Frederick, Maryland, United States of America; 5 Advanced Biomedical Computing Center, SAIC-Frederick, Inc., National Cancer Institute at Frederick, Frederick, Maryland, United States of America; 6 Sackler Institute for Comparative Genomics, American Museum of Natural History, New York, New York, United States of America; 7 Institute for Genomic Biology, University of Illinois at Urbana-Champaign, Urbana, Illinois, United States of America; Texas A&M University, United States of America

## Abstract

Conservation strategies for African elephants would be advanced by resolution of conflicting claims that they comprise one, two, three or four taxonomic groups, and by development of genetic markers that establish more incisively the provenance of confiscated ivory. We addressed these related issues by genotyping 555 elephants from across Africa with microsatellite markers, developing a method to identify those loci most effective at geographic assignment of elephants (or their ivory), and conducting novel analyses of continent-wide datasets of mitochondrial DNA. Results showed that nuclear genetic diversity was partitioned into two clusters, corresponding to African forest elephants (99.5% Cluster-1) and African savanna elephants (99.4% Cluster-2). Hybrid individuals were rare. In a comparison of basal forest “F” and savanna “S” mtDNA clade distributions to nuclear DNA partitions, forest elephant nuclear genotypes occurred only in populations in which S clade mtDNA was absent, suggesting that nuclear partitioning corresponds to the presence or absence of S clade mtDNA. We reanalyzed African elephant mtDNA sequences from 81 locales spanning the continent and discovered that S clade mtDNA was completely absent among elephants at all 30 sampled tropical forest locales. The distribution of savanna nuclear DNA and S clade mtDNA corresponded closely to range boundaries traditionally ascribed to the savanna elephant species based on habitat and morphology. Further, a reanalysis of nuclear genetic assignment results suggested that West African elephants do not comprise a distinct third species. Finally, we show that some DNA markers will be more useful than others for determining the geographic origins of illegal ivory. These findings resolve the apparent incongruence between mtDNA and nuclear genetic patterns that has confounded the taxonomy of African elephants, affirm the limitations of using mtDNA patterns to infer elephant systematics or population structure, and strongly support the existence of two elephant species in Africa.

## Introduction

Central to the successful management of endangered taxa is determination of whether their populations comprise one or more species. Molecular methods can play a major role in establishing the systematics of endangered taxa. Where more than one species is revealed, a separate conservation strategy would be justified for each. African elephants (genus *Loxodonta*) occupying tropical forest habitats were long considered to comprise a single species with elephants in other African habitats. Recent studies have renewed debate about their taxonomy, after both morphological and nuclear DNA analyses suggested that African elephants form two distinct species separated by a relatively narrow hybrid zone [1]. Skull measurements from 295 elephants of known provenance suggested that forest and savanna elephants fall into two morphologically distinct species [2], [3]. Nuclear DNA analyses using both slower-evolving nuclear sequences [4], [5], [6] and more rapidly evolving microsatellite loci [7] have provided concordant evidence that forest and savanna elephants are distinct species [4], [6], [8], [9] that are as divergent genetically as Asian elephants are from mammoths [5]. Few morphological intermediates [3], [10] and nuclear genetic hybrids [4], [6], [7] between forest and savanna elephants have been detected, primarily in a zone of mixed forest-savanna habitat that surrounds the tropical forests of Africa [1].

The deeper relationships present among mtDNA lineages have also been determined in African elephants [4], [11], [12], [13]. While two highly divergent mtDNA clades are present, a non-monophyletic pattern was revealed that did not cleanly separate forest and savanna elephants. Studies basing their conclusions primarily or completely on mtDNA data have inferred conflicting conclusions about African elephant systematics. Debruyne (2005) has stated that “the only defendable attitude assumes that Africa harbors a single species of elephant” [12]. Eggert et al. (2002) [14], [15] have hypothesized that the elephants “of west Africa belong to a newly recognized and yet to be formally named species” [14]. Johnson et al. (2007) have stated that “the classification of species into savannah and forest may not reflect their evolutionary history but simply the habitat in which they currently exist”; and that Africa's elephants fall into three or four “groups” with “implications for taxonomy” [16]. By contrast, analyzing both mtDNA and nuclear genetic patterns within the same individuals and populations, Roca et al. (2005) [4] discovered that the non-monophyletic mtDNA pattern was strongly incongruent with patterns present among nuclear markers (characterized as “cyto[or mito]-nuclear genomic dissociation”), and therefore did not detract from the two-species model for living *Loxodonta* taxa [1].

These sometimes strikingly opposed interpretations of molecular data are influenced by the types of genetic markers used [1]. Thus, despite exceptionally intensive genetic analysis, opposing taxonomic designations of African elephants persist to date, and the African Elephant Specialist Group and Species Survival Commission of the International Union for the Conservation of Nature (IUCN) have cited the multiplicity of taxonomic interpretations of genetic data as justification for its decision to “continue to treat African elephants as a single species” [17]. A 2011 published survey of conservation priorities among afrotherian mammals concluded that resolution of the relationships and diversity among African elephants constitutes an “urgent priority” given “the important conservation implications of elephant taxonomy” [18].

In this study, we sought to resolve these apparent conflicts by further examining nuclear and mtDNA patterns among savanna elephants using a novel set of short tandem repeat (STR) loci in African forest and savanna elephants that had previously been typed for mtDNA. Employing a Bayesian clustering approach, we sought to better quantify the degree and geographic extent of hybridization based on these STR loci. We examined the relationships between mtDNA and nuclear patterns in these elephants and determined what these patterns imply about evolutionary relationships of almost a thousand African elephants from 81 geographic locales for which mtDNA sequence had been previously generated. We determined whether geographically mapping the mtDNA and nuclear genetic partitions among the elephants of Africa might provide additional insights into resolving the differing assertions regarding their taxonomic status. We found that our novel approach to mtDNA analysis could reconcile the apparent conflict previously reported between mitochondrial and nuclear phylogenies in African elephants [12].

Based on this systematic clarification, we further sought to improve upon the other major area in which genetics can contribute to African elephant conservation: the forensic analysis of ivory samples. Although the Convention on International Trade in Endangered Species (CITES) banned the ivory trade in 1989, large numbers of elephants continue to be poached for their tusks, and the illegal trade in ivory is a major threat to their conservation [19]. Since ivory is often confiscated in markets far from the locations where elephants are poached, DNA markers have been used to identify the source population of confiscated ivory [20], [21]. Wasser and colleagues have successfully extracted DNA from small amounts of elephant ivory [22], [23], and applied spatial smoothing methods to allele frequencies of STRs in order to assign ivory to its source. Using this approach, they have genotyped tusks of smuggled ivory and assigned them as originating within a particular range country [24]. The success of these methods is encouraging, though further enhancement of the accuracy and precision of assignment would be desirable [25], [26]. We therefore also sought to improve DNA-based methods for tracking ivory by developing a means of quantifying the effectiveness of each of our STR loci for assigning elephants to their place of origin, and to identify those STR loci most and least effective at geographic assignment.

## Results

**Figure 1:**
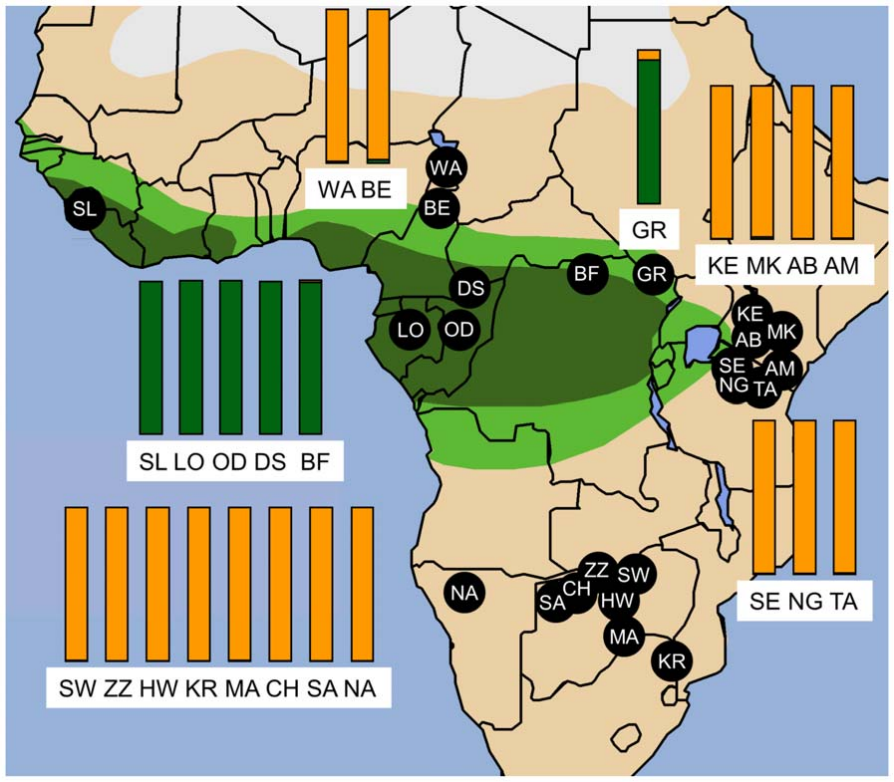
Location map and nuclear genetic clustering of sampled elephant populations in Africa. The map portrays the extent of tropical forest (dark green), and the forest-savanna transition zone (light green) [28]. Bar plots for each locale represent the average contribution to the genotypes of elephants made by forest (green) or savanna (orange) elephants, as partitioned using the program STRUCTURE (*K* = 2) using data from 11 microsatellite loci (see Figure 2). STRUCTURE cluster 1 (green) comprised 99.5% of forest populations and 0.6% of savanna populations. Cluster 2 (orange) comprised 0.5% of forest populations and 99.4% of savanna populations. Sampling locations in forest habitats were: DS-Dzanga Sangha, Central African Republic; OD-Odzala, Congo (Brazzaville); BF-Bili Forest, Congo (Kinshasa); LO-Lope, Gabon; and SL-Sierra Leone (one zoo individual). Savanna locations: CH-Chobe, MA-Mashatu, SA-Savuti in Botswana; BE-Benoue, WA-Waza in Cameroon; AB-Aberdares, AM-Amboseli, KE-Central Kenya/Laikipia, MK-Mount Kenya in Kenya; NA-Northern Namibia/Etosha; KR-Kruger in South Africa; NG-Ngorongoro, SE-Serengeti, TA-Tarangire in Tanzania; HW-Hwange, SW-Sengwa, ZZ-Zambezi in Zimbabwe. GR-Garamba is located in the Guinea-Congolian/Sudanian transition zone of vegetation in D.R. Congo that historically included a mixture of forest and secondary grasslands [28] suitable for both African elephant groups [3].

Samples of African elephants collected primarily by dart biopsy, as previously described [11], [27], were grouped by geographic location of collection (Figure 1). Samples were grouped into 17 savanna locales and 5 forest locales, all except for the elephants of Garamba, which are from a region historically of mixed forest and savanna habitats [28] where both types of elephants have been reported based on morphology [3], [10], and where hybrids have been detected based on morphology [10] or nuclear genotypes [4], [6], [7]. Samples recently collected included forest elephants from the Bili Forest in the Democratic Republic of Congo, a location previously not included in elephant genetic studies; and a forest elephant sample from the Paris Zoo (France) of Sierra Leone origin (Figure 1) [29]. We also report from previously sampled locations [7] the STR genotypes of a much larger number of individuals. A total of 555 African elephant individuals (75 forest, 19 Garamba and 461 savanna elephants) were successfully genotyped, along with 9 Asian elephants (*Elephas maximus*), using fifteen microsatellite loci, including newly developed loci (Tables S1, S2), with a minimum of 11 loci used for each of the analyses. Summary statistics for the loci are shown in Table S2.

### Genetic separation between forest and savanna African elephants

**Figure 2:**
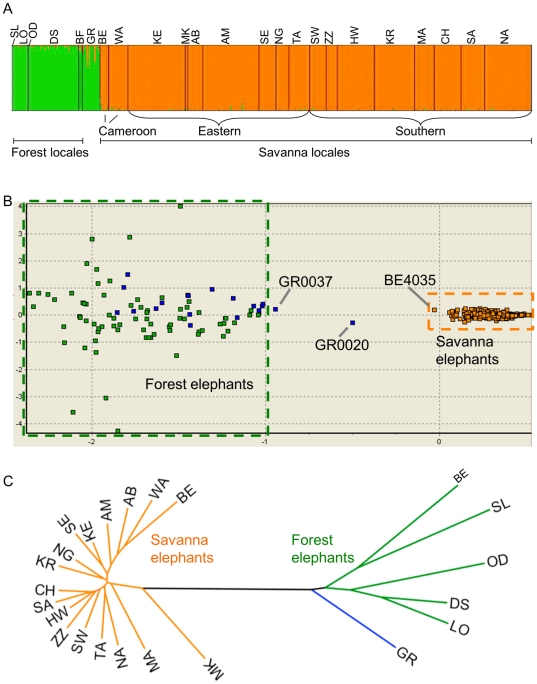
Species subdivisions and population substructure among African elephants. (A) Distinctiveness of forest and savanna elephant species and the pattern of hybridization between forest and savanna elephants was demonstrated using the program STRUCTURE, which applies a model-based clustering algorithm to identify subgroups that have distinctive allele frequencies [30]. The two partitions correspond to African forest elephant ancestry (green) and African savanna elephant ancestry (orange), and each is confined almost exclusively (≥99.4%) to locales in, respectively, tropical forest and non-tropical forest habitats (Figure 1). Garamba (GR) is the only population that spans an intermediate habitat zone containing both forest and savanna vegetation, and both types of elephants [3], [28]. (B) A factorial correspondence analysis (FCA) implemented using the software GENETIX [35] reveals the distinctiveness of forest and savanna elephants, with a very limited degree of hybridization. A total of 555 African elephant individuals (75 forest, 19 Garamba, and 461 savanna elephants) were used in the analysis. The two major axes determined by the FCA were used; plotted on the *x*- and *y*-axes, respectively accounting for 6.68% and 2.26% of the total variability. Savanna elephants formed a group distinct from the group formed by forest plus Garamba elephants. With only one exception (GR0020), hybrid elephants do not occupy the middle space between forest and savanna elephants; rather, they tend to show only a low level of admixture [4]. (C) Neighbor-joining phylogram depicting the genetic relationships among elephants by locale, based on the STR data using the chord distance. Elephants from savanna (orange) and forest (green) locales form distinct groups. Locale abbreviations are as in Figure 1.

Bayesian clustering analysis was performed on the microsatellite data using the program STRUCTURE [30], with strong evidence for *K* = 2 clusters among African elephants (Table S3) [31]. With *K* = 2, the clusters showed a geographic split that corresponded closely to the split between forest and savanna elephants (Figures 1, 2), in agreement with previous studies of morphology and nuclear genetic markers [1], [2], [3], [4], [6], [7], [13], [32], [33]. Every elephant from a tropical forest locale was identified as completely or primarily of forest elephant ancestry (Cluster 1 in green), while not a single elephant assigned primarily or completely to cluster 1 was from a location outside of tropical forest or mixed habitat (Figure 2). Likewise, every individual from a locale outside the tropical forest range was identified as primarily or completely savanna-elephant like (Cluster 2 in orange) in genotype, while elephants assigned predominantly or completely to Cluster 2 were all from locales outside the tropical forest range (Figure 2). There were no exceptions.

The overall contributions of Clusters 1 and 2 (forest elephant and savanna elephant genotypes, respectively) to the elephant population at each locale are shown in Figure 1. Across all elephants at tropical forest locales, the estimated percentage assignment to the forest elephant cluster averaged 99.5%. Among all savanna elephants, assignment to the savanna elephant cluster averaged 99.4%. In some cases, a low level of ancestry was assigned to the cluster of the other species. Some of these represent the few individuals that previous nuclear sequences have shown to be hybrids [4], [6], [7]. For others, the small proportion partitioned to the other species may reflect lower limits of resolution for the software STRUCTURE.

One of our locales, Garamba, is in a region of the Democratic Republic of Congo that historically included both forest and savanna habitats [28], [34]. Previous genetic studies also established the presence of hybrid nuclear genotypes in our Garamba samples [4], [6], [7]. This finding is supported by the current analysis, which indicated that Garamba has the greatest degree of nuclear genetic admixture detected between forest and savanna elephants (Figure 1), although with a much larger proportion of ancestry partitioned to the forest cluster (90.8%; Figure 1). Individual hybrids previously identified using nuclear gene sequences (notably in Bénoué and Garamba) [4], [6] are also apparent in the STRUCTURE analysis (Figure 2). No genetic hybrid has ever been previously detected among elephants in locations deep in the tropical forest [4], [6], [7], although among the savanna elephants two individuals in Cameroon had been identified as hybrids [4]. Overall, the elephants in Cameroon did demonstrate a slightly lower overall assignment to the savanna cluster than other savanna locales, with 95.6% assignment to the savanna cluster for Bénoué elephants, and 99.7% assignment for Waza; although even in Cameroon savannas, the forest elephant genetic contribution was quite limited (Figures 1 and 2). Contrary to the hypothesis that forest-savanna elephant hybrids are both common and widespread [12], [16] these findings support the view that hybrids are rare and limited in geographic distribution (Figures 1, 2) [1], as the overall proportion of genotypes inferred to derive from admixture with the other species was less than one percent for both forest and savanna elephants.

A factorial correspondence analysis (FCA) implemented using the software GENETIX [35] also demonstrated distinctiveness between forest and savanna elephants (Figure 2). The low degree of hybridization, even among the few populations that contain hybrids, was also evident in this analysis. Figure 2 shows the representation of the two major axes determined by the FCA in a combined analysis of all African elephants. The 461 savanna elephants formed a distinct group from 75 forest elephants. The apparent genetic diversity of the forest/Garamba elephants is noteworthy (and likely accounts for much of the variability in the FCA), especially given that many more savanna elephants from a wider geographic range were used for the analysis, and given that ascertainment bias would tend to increase the relative diversity of the savanna elephants in which most of the STR markers were selected for their polymorphism (Table S1). The lack of true genetic intermediates was striking in the FCA. With only one exception (GR0020), hybrid elephants did not occupy the middle space between forest and savanna species; rather, they tended to show only a low level of admixture, even in the hybrid zone of Garamba (Figure 2). Thus hybridization of forest and savanna elephants has not led to a “hybrid swarm” condition in which some populations are comprised primarily of genetic hybrids between the two species.

Genetic relationships among different locales were also inferred from the STR data using the chord distance. The elephants from savanna and forest locales formed two distinctive phylogenetic clusters (Figure 2). Likewise, our STRUCTURE results were not different after (1) the number of individual savanna elephants was randomly reduced to equal the number of forest elephants (run multiple times with different sets of savanna elephants); (2) the number of forest and savanna elephant individuals was randomly trimmed to each equal the number of Garamba elephants (run multiple times with different sets of forest and savanna elephants); or (3) the number of STR loci genotyped was increased to 27 for a subset of 35 forest, 9 Garamba, and 142 savanna elephants. Consequently, our results were robust across methodologies and across different resampling schemes of individuals and loci.

### Geographic distribution of nuclear and mtDNA partitions

**Figure 3:**
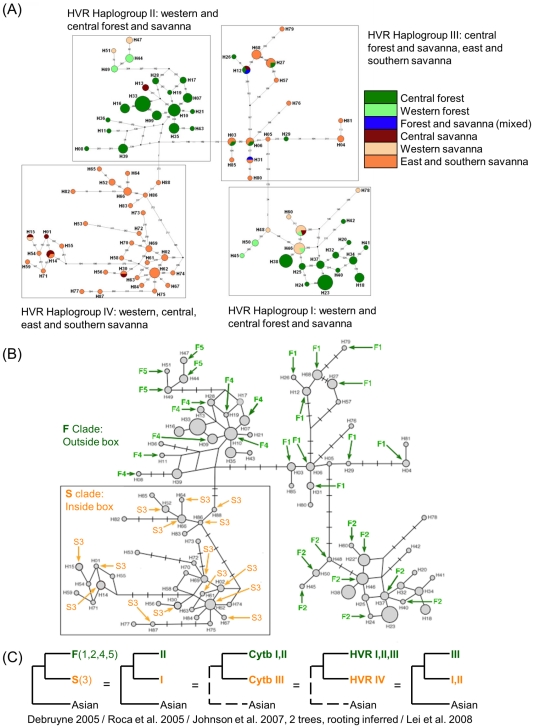
Relationship of the four groups identified by Johnson and colleagues to the F and S mtDNA clades and subclades described by other researchers. (A) Johnson et al. (2007) [16] proposed dividing elephants into four novel “groups” based on an unrooted network using the D-loop hypervariable region of mtDNA (reproduced under a Creative Commons Attribution license, from BioMed Central Ltd). (B) We identified the mtDNA haplotypes of Debruyne (2005) [12] and Eggert et al. (2002) [15] within the Johnson et al. (2007) [16] network, finding that there was considerable overlap between the mtDNA diversity reported by Johnson et al. (2007) and the diversity reported by the prior studies. Debruyne (2005) [12] had reported strong support for a separation of African elephant mtDNA haplotypes into two deep subdivisions, designated mtDNA clades “F” and “S”, subsequently verified by two other researcher teams [4], [13]. Debruyne (2005) [12] established further subdivisions within the F clade (designated F1, F2, F4 and F5; the S clade was also designated S3 with no further subdivisions) [12], [15]. Three of the groups identified by Johnson et al. (2007) [16] corresponded to subclades of the F clade previously identified by Debruyne (2005) [12]; the other corresponded to the S clade. (C) Across the previously reported mtDNA surveys of African elephants, sufficient overlap existed among sequences and individuals for mtDNA relationships to be established across all of the studies. Cladograms show the naming conventions used for S clade and F clade mtDNAs across different studies [4], [12], [13], [16], including that of Johnson et al. (2007), which had incorporated the sequences of Eggert et al. (2002) [15] and Nyakaana et al. (2002) [37]. The “clade F” identified by Debruyne (2005) [12] proved synonymous with the other clade designations shown in green; the “clade S” identified by Debruyne (2005) [12] proved synonymous with the other clade designations shown in orange [4], [12], [13], [16]. Supplementary Figure S2 details the overlapping informative sequences that established the relationships shown across the datasets.

The mtDNA lineages of African elephants have been studied by a number of researchers who used different designations for mtDNA haplogroups, and who often compared different regions of the mtDNA genome [4], [12], [13], [15], [16], [36], [37]. We were able to combine the results of all of these studies, including that of Johnson et al. (2007) [16] which had included the sequences of Eggert et al. (2002) [15] and Nyakaana et al. (2002) [37] and others, by comparing overlapping fragments of mtDNA across the studies. Given the priority of Debruyne's (2005) [12] publication (which established with strong bootstrap support the deepest relationships among haplogroups), the current study employs the clade “S” and clade “F” designations of Debruyne (2005) for the two most deeply rooted mtDNA clades. Since mtDNA is non-recombinant, all genes or regions in the mtDNA genome have followed the same evolutionary trajectory [9]; and since some studies had sequenced individual elephants for two or more mtDNA regions [12], [13], it was possible to discover the equivalent designations used for the F and S clades across the other elephant mtDNA studies (Figure 3; Figure S1), even across studies that used non-overlapping regions of the mtDNA genome (see Figure S2 for details). For example, Roca et al. (2005) [4] had sequenced a portion of the mtDNA *ND5* gene and detected two deep clades, designated I and II (Figure 3). Debruyne (2005) also detected two deep clades, by sequencing the *CYTB* mtDNA gene, calling them Clades S and F. Sequences from both studies were compared to those of Lei et al. (2008), who had sequenced individual elephants for longer mtDNA regions that overlapped with the sequences both of Debruyne (2005) and of Roca et al. (2005) (Figure S2) [4], [12], [13]. Using this system, designations for the equivalent clades across studies were identified. The mtDNA clade designations used by all other elephant mtDNA surveys for clades or “groups” corresponding to the F/S clade nomenclature of Debruyne (2005) [12] are shown in Figure 3.

In the most recent of these surveys, Johnson et al. (2007) [16], reported the presence of four taxonomic groups of elephants in Africa. They hypothesized that they had uncovered the presence of new genetic groupings of African elephants due to their collection of elephant samples from previously unexamined locations. In our attempt to identify haplogroup synonyms across various studies, we identified the elephants of Debruyne (2005) [12] and Eggert et al. (2002) [15] within the network of Johnson et al. (2007) [16] (Figure 3). We found that the elephant groups of Johnson et al. (2007) were synonymous with five subclades of the basal F and S clades previously identified by Debruyne (2005) [12], with the subclades designated F4 and F5 by Debruyne (2005) [12] consolidated into “Group II” by Johnson et al. (2007) [16] (Figure 3 and Figure S1). Thus, while recognizing the importance of the intensive geographic sampling conducted by Johnson et al. (2007) [16], we consider that the best interpretation of the mtDNA network generated by Johnson et al. (2007) would not be that previously undetected genetic groupings of elephants had been found, but rather that one of their groups corresponded to the mtDNA S clade, while each of the other groups of Johnson et al. (2007) corresponded to subdivisions of the mtDNA F clade identified previously by Debruyne (2005) [12] and Eggert et al. (2002) [15] (Figure 3).

**Figure 4:**
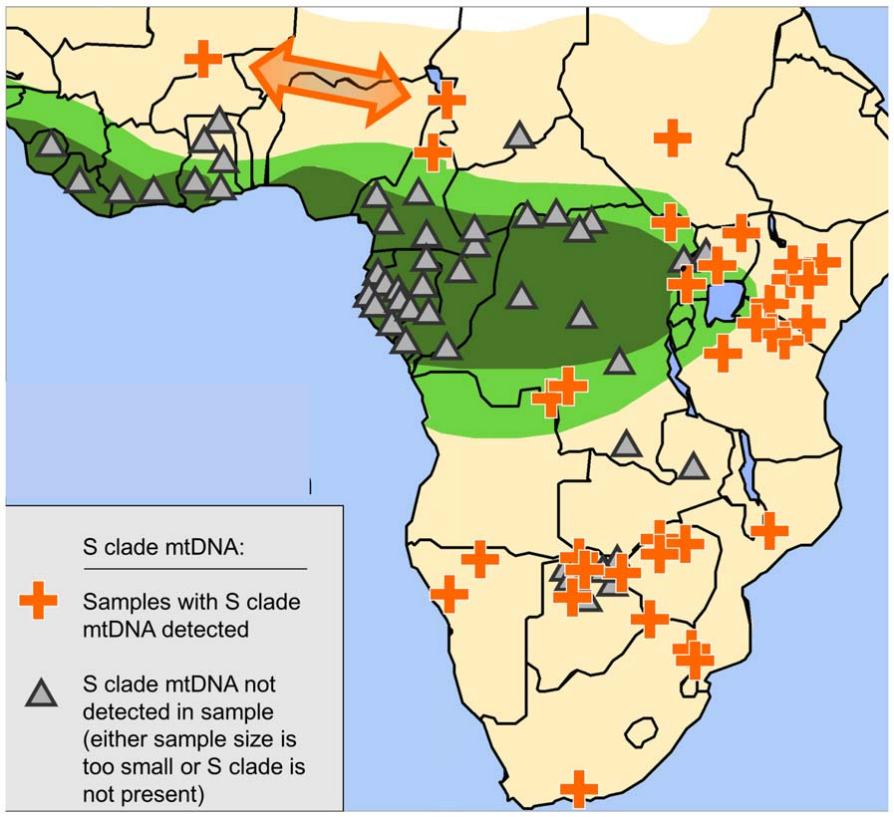
The distribution of “S” clade mtDNA across the African continent corresponds to the distribution of savanna elephants. The map shows the distribution of S clade mtDNA across Africa, using results reported by all previous continent-wide studies of elephant mtDNA in Africa [4], [12], [13], [15], [16], [36], [37], with results shown by location. A plus sign (orange) on the map indicates that S clade mtDNA was present at the location (whether or not F clade mtDNA was also detected). A triangle indicates that S clade was not detected in the sample. S clade mtDNA was completely absent across the entire range of forest elephants at 30 tropical forest locales (dark green) across West and Central Africa. By contrast, at some savanna locales where S clade was not detected in some samples, such as Botswana, the apparent absence of S clade was likely due to an insufficient numbers of elephants being sampled, since other nearby samples did detect S clade. The presence or absence of S clade mtDNA was found to be highly significantly associated with type of habitat (tropical forest vs. other or mixed habitats; Fisher's exact test, two-tailed *p*<10^−4^). The distribution of S clade closely corresponds to the geographic distribution of savanna elephant habitat, morphology and nuclear genotypes [2], [3], [4], [6], [7]. The arrow indicates that the mtDNA S clade haplogroup was also present in West African savanna elephant populations. Further information on sample types and locations is supplied in Supplementary Figure S3.

**Figure 5:**
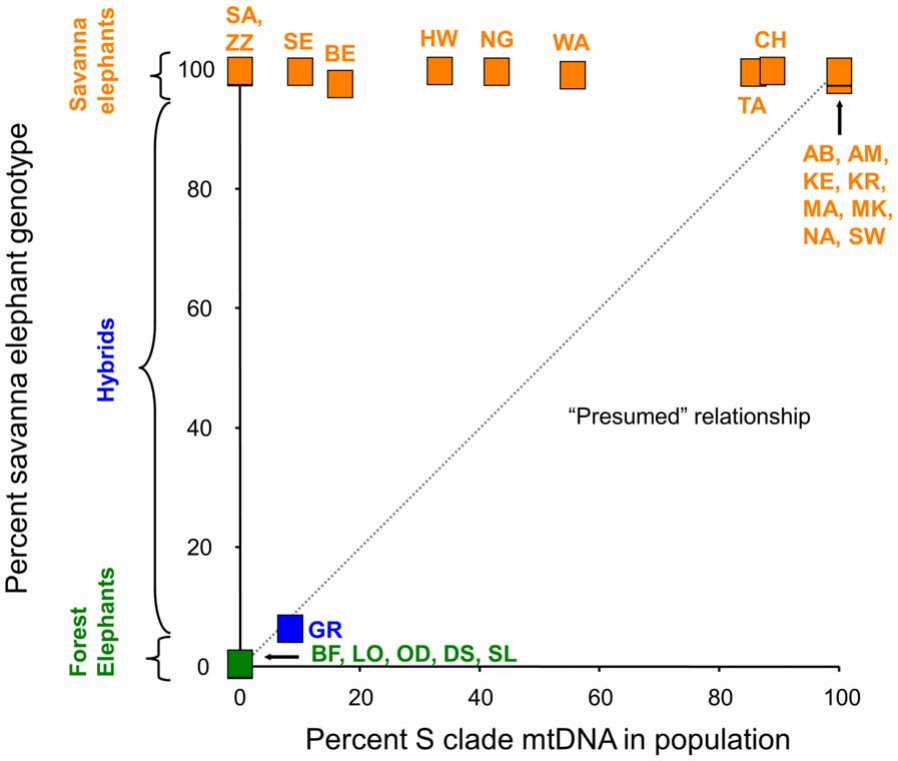
Partitioning of forest and savanna elephant nuclear genotypes and mtDNA haplotypes at each African locale. For each location, the *x*-axis shows the proportion of mtDNA from the S clade or savanna clade of mtDNA [4]. The *y*-axis shows the proportion of the total population that corresponds to cluster 2 (savanna elephant nuclear genotype) of the STRUCTURE analysis. Savanna elephant locales (orange) all have a very high proportion of savanna elephant nuclear genotypes. By contrast, the proportion of savanna elephant S clade mtDNA varies from 100% down to 0% among the savanna locales, with no effect on nuclear genotypes. Remarkably, the proportion of S clade mtDNA varies greatly on a local level (e.g., CH-Chobe and SA-Savuti are geographically adjacent locales), calling into question attempts to infer the population structure of African elephants using only mtDNA. Forest elephant (green) locales show little or no nuclear genetic contribution from savanna elephants. None of the forest elephants carry a savanna elephant or S clade mtDNA [4], [29], in this limited number of sampling locales (see Figure 4 for a more extensive survey of mtDNA data). The dashed line shows the “expected” relationship, if an assumption is made that the proportions of forest and savanna elephant nuclear genotypes in a population should reflect the proportion of F and S clade mtDNA haplotypes, respectively, in the population [12], [16]. This “expected” relationship forms the basis for statements that forest and savanna elephants currently have a geographically large hybrid zone with large numbers of hybrids present across Africa [12], [16]. Our current data suggests that such inferences are not valid since mtDNA patterns do not reflect population genotypes. Locale names are abbreviated as in Figure 1; we show the only mixed habitat locale (GR-Garamba) in blue.

Having established the relationship of mtDNA designations across studies, we combined all of their results, assessing F and S clade mtDNA data on elephants from 81 locations across Africa (Figure 4; Figure S3) [4], [12], [13], [15], [16], [36], [37]. The conclusion drawn from this combined dataset was remarkable: not a single elephant from the African tropical forests carried S clade mtDNA (Figure 4). The combined results surveyed at least 30 tropical forest locations across both Guinean and Congolian forest blocks (Figure 4, Figure S3), yet S clade was not detected at any tropical forest locales. This is especially remarkable given that three independent surveys have each reported, by contrast, that about 20% of Africa's savanna elephants carry forest elephant (F clade) mtDNA (Figures 3, 5) [4], [12], [13].

Although S clade was not detected in all savanna locales, in many cases, the apparent absence of S clade at a locale was likely due to an insufficient number of elephants being sampled, since samples from nearby savanna regions did detect S clade (e.g., in northern Botswana; Figure 4). Some previous studies had analyzed mtDNA haplogroups before Debruyne (2005) identified the basal F and S clades [15], [37]; others did not consider whether their mtDNA haplogroups comprised subsets of the F or S clade [16]. Thus the pattern we are demonstrating for the distribution of mtDNA S clade has not been reported previously with this degree of geographic sampling. Our approach to mtDNA phylogeography (Figure 4) produced another notable result: the presence of S clade mtDNA closely corresponds to the range boundaries traditionally ascribed to the savanna elephant species based on nuclear DNA (Figure 1), morphology and habitat [1], [2], [3], [4], [6], [7], [13], [24], [25], [26], [32], [33], [38], [39].

This helps to reconcile the apparent conflict previously reported between phylogeographic patterns of mtDNA in African elephants and the very different geographic patterns in morphology and nuclear genotypes [12]. For mtDNA the presence or absence of S clade corresponded to the geographic pattern previously reported using nuclear and morphological markers [3], [6]. A contingency table was analyzed, comparing the presence (vs. lack of detection) of S clade mtDNA to the type of habitat at a locale (tropical forest vs. other or mixed habitats). Since 30 tropical forest locales lacked elephants carrying S-clade mtDNA, while S clade was detected in 34 of 51 non-tropical-forest samples, the association was found to be extremely significant (Fisher's exact test, two-tailed *p*<10^−4^).

Genetic studies of African elephants have detected mito-nuclear discordance [4], in which patterns inferred using mtDNA markers do not correspond to those found using nuclear markers in the same forest and savanna elephants [4], [11], [12], [13]. We therefore compared the partitioning of elephant nuclear genotypes by the program STRUCTURE with the pattern of mtDNA present in the same elephant individuals and locales [4]. In Figure 5, each African elephant locale is plotted with the *x*-axis showing the proportion of mtDNA haplotypes that belong to the “S clade” or savanna clade of mtDNA [4], while the *y*-axis shows the proportion of the nuclear genotypes assigned to the savanna elephant cluster by STRUCTURE (see Figure 1). Although savanna elephant locales all had an overwhelmingly high proportion of savanna elephant nuclear genotypes, the proportion of savanna clade mtDNA (S clade) carried by the elephants at savanna locales can range from 100% down to 0% (Figure 5) [4]. In fact, the proportion of savanna elephant nuclear genotypes carried by savanna elephants appeared to have no relationship to the proportion of S clade savanna mtDNA present in the population (Figure 5). By contrast, while forest elephant locales showed little or no nuclear genetic contribution from savanna elephants, they also carry no S clade mtDNA (Figure 5) [4]. Previous studies have made the suggestion that forest and savanna elephants have a geographically expansive hybrid zone with large numbers of hybrids present across Africa [12], [16], based on the often unstated assumption that the proportions of forest-savanna nuclear markers in a population likely reflect the proportion of F and S clade mtDNA markers. In Figure 5, the dashed line shows this “presumed” relationship if the proportions of S and F clade mtDNAs are assumed to serve as accurate estimators of the overall contribution of savanna and forest lineages to the nuclear genetic makeup of the elephants. If this were the case, many savanna populations should have a high proportion of forest elephant admixture. Instead, the actual data showed that no relationship existed for savanna elephants between the proportion of F to S clade mtDNA and the degree of hybridization present in the locales. Thus the suggestion that African regions that include both F clade and S clade elephants represent geographically extensive hybrid zones in which both forest and savanna elephants are present and the populations are a mixture of the two [12], [16] is contradicted by the current data for Cameroon, Southern and Eastern Africa (Figure 5). Since the lack of association between mtDNA and nuclear patterns holds true for these three regions, one may also question the validity of taxonomic and population genetic inferences drawn from analogous mtDNA patterns in West Africa.

### Nuclear genetic similarity between Central and West African elephants

The Guinean forest block in West Africa is currently not contiguous with the Congolian forest block in Central Africa [28], [40]. One genetic study that conducted extensive sampling of elephants in West Africa hypothesized that West African elephants may comprise a third species of elephant, separate from forest and savanna elephants in the rest of Africa [14], [15]. The single elephant in our sample from a West African locale in the Guinean forest block, for which the mtDNA haplotype [29] further established its West African origin [15], clustered with the Central African elephants from the Congolian forest block (Figures 1 and 2), even when greater numbers of genetic clusters (*K*) were considered in the STRUCTURE analysis (not shown); and this individual has previously been found to have nuclear sequences identical to that of some Central African forest elephants (though distinct from that of any savanna elephants) [32]. Of course, a single sample would be insufficient to reach conclusions about the affinities of elephants in West Africa.

We therefore considered an alternative if indirect approach to assessing the genetic similarity between West and Central African elephants. There has been one previous study that examined a large number of nuclear genetic markers among elephants in both West and Central Africa, which reported on the use of these markers in geographic assignment tests of elephants [26], but without additional analyses of genetic structure. These and other previous genetic assignment tests have been able to identify individuals as being Asian, African savanna, or African forest elephants, with 100% success [7], [26]. The success of these assignment methods was seen as supporting the division of African elephants into forest and savanna elephant species, since members of one species were never incorrectly assigned to the other species [7], [26]. The assignment tests reflect the nearly complete lack of gene flow between forest and savanna elephants that has also been inferred using other nuclear loci [4], [6]. Since lack of gene flow between populations would be an indication that speciation has occurred, we re-examined this previously published data [26], comparing assignment success between West and Central African elephant locations to determine whether this would provide support for the hypothesis that they comprise distinct species.

**Figure 6:**
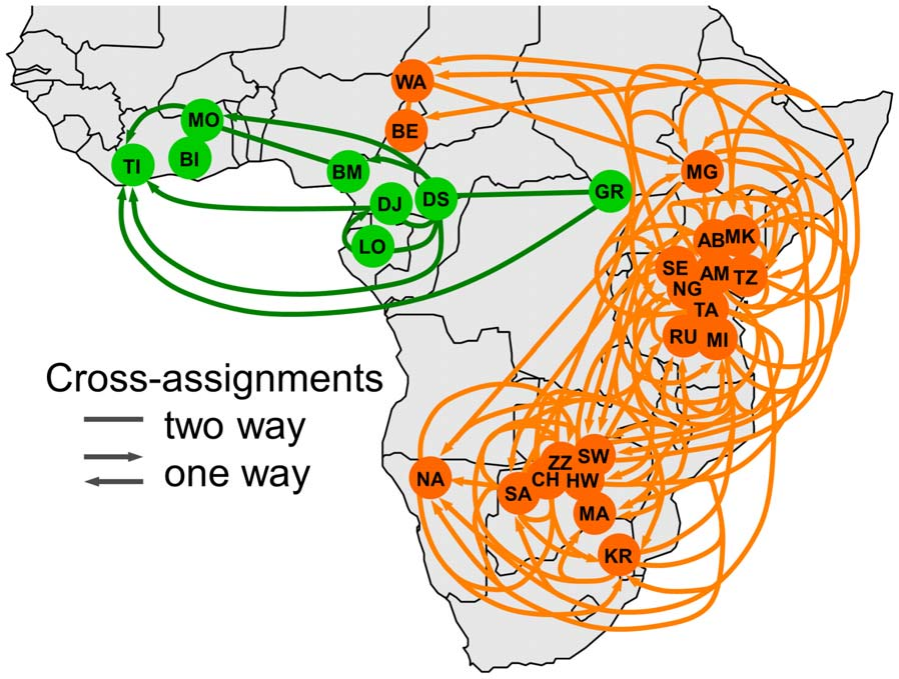
West African elephants–a distinct third species? This map, depicting the mis-assignment of elephants to the wrong locales by an STR-based assignment method, does not lend support to the hypothesis that West African elephants are a distinct species. Assignment data is from Wasser et al. [26], but plotted geographically with forest elephant locales in green and savanna locales in orange. Arrowheads indicate the direction of misassignment (e.g., elephants in GR were assigned wrongly to TI); lines without arrowheads indicate that misassignments occurred in both directions. Each misassignment would be an indication of genetic similarity between elephants at the two locales. Among the hundreds of elephants assigned using STRs [26], there is no overlap in cross-assignments between forest and savanna elephant groups, even in cases where some forest and savanna elephant locales were geographically much closer between than within species (e.g. BE is closer to DS than to most other savanna locales); nor did misassignment ever occur between Asian and African elephants. By contrast, a very high degree of cross-assignment is evident between locales in West Africa and those in Central Africa, an indication of substantial genetic similarity between elephants in the two forest blocks, which fails to provide support for the hypothesis that a distinct elephant species inhabits West Africa [14], [15]. Locales are as in Figure 1, with additional locales: BI-Bia, BM-Banyang Mbo, DJ-Dja, MG-Mago, MI-Mikumi, MO-Mole, RU-Ruaha, TI-Tai, TZ-Tsavo [26].

Figure 6 shows a map with all locations used in the prior assignment study [26], including many locales outside of those shown in Figure 1, and including a substantial number of sites in both West and Central Africa. As an indirect measure of genetic similarity, we plotted the misassignment of elephants between one location and another, which is an indication of genetic similarity between elephants at two locales. Lines connecting the locales indicate cases where at least one elephant of known provenance was assigned to the wrong locale. What is striking is that among the hundreds of elephants assigned to a location using nuclear genotypes [7], [26], not a single savanna elephant has been assigned to a forest location [26], even in cases where forest and savanna locales were geographically much closer between than within species. However there were many mis-assignments between two savanna or between two central forest locales (Figure 6). Given the complete success in assignment of elephants as either savanna (orange) or forest (green) elephants, the substantial degree of misassignment between Congolian and Guinean block forest elephants (Figure 6) suggested that nuclear genetic similarity exists between forest elephants in the two forest blocks. The genetic similarity implied by the misassignment of elephants between the two forest blocks fails to provide support for the hypothesis that the elephants of West Africa comprise a distinct species [14].

### Quantifying STR loci for their utility in assigning geographic provenance

Besides taxonomy and population structure, the other major area in which genetics can contribute to African elephant conservation is in the geographic assignment of ivory samples [20], [21], [22], [23], [24], [26]. Current geographic assignment methods for elephants and ivory that use STRs may sometimes be imprecise or inaccurate (Figure 6) [7], [24], [25], [26]. The mis-assignments depicted in Figure 6, for example, show that room exists for improvement of genetic markers to establish the provenance of elephants (and their ivory). Furthermore, the considerable variation in allelic size range for different STR loci used in the current study (Table S2) suggested that some STR loci would prove more effective than others for genetically assigning an elephant to its geographic origin. While previous studies have shown that STR genotypes can be used to assign ivory to its geographic region of origin [7], [24], [25], [26], they have not attempted to rate the effectiveness of individual STR loci in assignment studies. We therefore adopted methods used in admixture mapping of human disease genes, for which genetic markers have been identified that have a high ability to distinguish among human lineages of different geographic origin [41], [42], [43], [44], to determine the utility of each elephant STR locus for geographic assignment.

In order to quantify the ability of different genetic markers to assign the provenance of elephants, the Shannon Information Content (SIC) [45] of each microsatellite locus was estimated. The SIC is often preferred over other measures of informativeness because many other measures do not consider the influence of centrality, whereby alleles that are absent (or nearly so) in one population will be more informative than those common in both populations [41], [42]. The SIC quantifies the informativeness of the marker or set of markers in determining the ancestral state (or source of origin) of the loci [41], [42]. In the case of a biallelic locus such as a microsatellite in an elephant sample of unknown provenance, it is necessary to know the frequency of the alleles in the parental populations from Africa, information that was provided by our current STR data.

**Figure 7:**
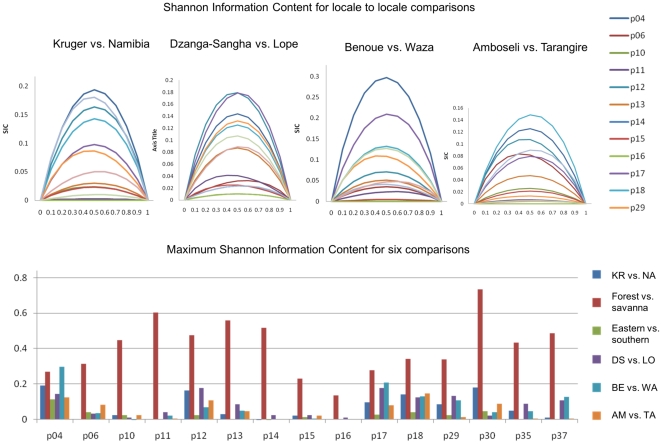
Quantification of the effectiveness of individual STR loci for establishing the provenance of African elephants. Each locus was tested for the ability to distinguish between forest and savanna elephants, between southern and eastern African savanna elephants, and between elephants at the two most populous locales within various regions. (Top) The Shannon Information Content (SIC) is plotted for four pairwise comparisons between locales for STR loci (note that the *y*-axis scale differs among comparisons). The maximum SIC does not always occur at the 0.5 admixture value since the locus may be more effective at distinguishing one population than the other. (Bottom) Most loci were better at distinguishing forest and savanna elephants than at distinguishing between two regional locales. Some STR loci, such as p18, are consistently informative for distinguishing between elephants from different locales; other loci such as p16 are consistently uninformative. This suggests that p18 should be used, and p16 replaced, when selecting a set of STR loci for use in establishing the origins of poached ivory. This quantitative approach comparing different STRs for their utility can be used to identify an improved set of STR loci that would enhance the ability to establish the origins of confiscated ivory [44].

The ability of each of our STR loci to differentiate among elephants from different locales is shown in Figure 7. Each locus was tested for the ability to distinguish between forest and savanna elephants, between southern and eastern African savanna elephants, and between elephants at the two most sampled locales within various regions. Highly informative loci for a given pair of populations had the highest values of SIC, for example, p04 in the Kruger-Namibia comparison (Figure 7, top). For almost all markers (except p04), the highest information content was present in the savanna-forest comparison, with the next most informative comparisons coming in a distant second (Figure 7, bottom). Most maximum SIC values coincided with the 50% admixture proportion overall, but some individual values deviated. For example, the maximum SIC for p11 in the Kruger-Namibia comparison (the gray line, second in height) occurred slightly towards the left, thus the information content is not the same. The maximum SIC does not always occur at the 0.5 admixture value since a locus may be more effective at distinguishing one population than the other. For example, if the major allele frequency is about 50% in population one, but 100% in population two, it would be more effective to use the given marker to identify individuals from population one in population two than vice versa.

As has been shown in the case of human STRs [44], our results suggested that replacement of relatively uninformative loci by highly informative loci could produce a set of markers with an ability much greater than that of the current set of randomly chosen markers to establish the provenance of illegally poached ivory. For example, when we used 4 STRs with low SIC values (p11, p14, p15, p16) in a STRUCTURE analysis, they did not distinguish Kruger elephants at all from Namibian elephants, as 50% of each population fell into each of the partitions at *K* = 2 (using only these two populations). However, using 4 STRs with high SIC values (p12, p17, p18, p29) in a similar analysis did begin to differentiate elephants between these two locales: using only these two populations, at *K* = 2 the values for STRUCTURE partitions 1 and 2 were 0.36 and 0.64, respectively, for Kruger elephants; while they were 0.61 and 0.39, respectively, for Namibia elephants. This quantitative approach comparing different STRs for their SIC can thus identify an improved set of STR loci to utilize for the purpose of assigning the geographic provenance of elephants.

## Discussion

### Two species of elephant are present in Africa

Our new data and reanalyses of previous data reconciled the mtDNA phylogeographic patterns present among African elephants to the two-species division that is evident from nuclear DNA and morphological analyses. Previous studies found a deep and almost complete nuclear genetic separation of African elephants into forest and savanna species, with a few hybrids detected primarily in the regions where tropical forest and savanna habitats meet [4], [6], [7]. This genetic separation into forest and savanna elephant species has now been demonstrated using slower-evolving nuclear autosomal sequences, faster-evolving autosomal STRs, sequences of multiple X-linked loci, and Y-chromosome sequences, and was recently supported following comparison of hundreds of nuclear sequences across proboscideans [4], [5], [6], [7]. The lack of gene flow between forest and savanna populations, despite the existence of a hybrid zone, has especially supported their status as species under the biological species concept [46], [47], [48] (as previously discussed [1]): among 1764 savanna elephant X-chromosome sequences previously examined, 1762 (99.9%) had been found to be haplotypes not present among forest elephants, while not a single savanna elephant nuclear haplotype has ever been reported in a tropical forest elephant population [4]. Likewise, among Y-chromosome sequences examined in 205 male elephants, there had been only one hybrid exception to the otherwise complete separation into distinctive forest and savanna elephant clades [4].

The Bayesian STRUCTURE analysis conducted for the current study used a novel set of STR loci to support and extend these conclusions. Few forest–savanna elephant hybrids were identified by STRUCTURE, and for each species the proportion of admixture from the other species was inferred to be under one percent. Previous studies have convincingly reported data supporting the “polyphyly of the forest and savannah [mtDNA] haplotypes” [12] and “multiple refugial mitochondrial lineages” [16], and these observations are also in accordance with mtDNA patterns found among the elephants used for the current study [4]. However, based on these mtDNA patterns, it had been further hypothesized that “interbreeding has had a major impact on the reciprocal integrity of extant forest and savannah elephants” [12], and that “recurrent hybridization among them render[s] a simple forest/savannah elephant split inapplicable to modern African elephant populations” [16]. Yet these hypotheses regarding the overall genomic affinities of African elephants were not supported by the results of nuclear genotyping (Figures 1, 2, 5). The current genetic assignment and factorial correspondence analyses demonstrate nuclear genetic partitioning between forest and savanna elephants (Figures 1, 2), with nuclear admixture between species at under one percent, even in the face of widespread forest clade mtDNA introgression in the same savanna elephants (Figure 5) [4].

### Should incongruent mtDNA patterns in African elephants have been anticipated?

Our results also strongly support the observation that mtDNA patterns in elephants are incongruent with their overall population structure [1], [4], [13]. Before discussing the observed genetic patterns in detail, it is worth considering whether the phylogeographic patterns of mitochondrial genetic markers should have been expected to be incongruent with those of nuclear markers, given the social structure of elephants. Elephant females reaching maturity remain with their natal core social group or “herd” [49], [50]. Since females are not typically exchanged between herds [49], [50], mitochondrial gene flow between herds will be essentially nonexistent, and only changes in the geographic ranges of the core social groups would alter the phylogeography of mtDNA haplotypes. Since the mitochondrial genome is only transmitted maternally, there is an important consequence of the matrilineal and matrilocal structure of core social groups among elephants: the mitochondrial genome is necessarily coupled to the geographic range of the core social group. By contrast, males leave the natal herd and mediate gene flow between herds and across the African landscape [1], [49], [50], [51]. This is consistent with the observation that sex differences in dispersal tend to be high among long-lived, highly social, polygynous mammals [52], [53].

Since males can transmit every locus except mtDNA, the mitochondrial genome is subject to an extremely circumscribed evolutionary trajectory very different from that affecting every other genetic locus [1], [54]. For example, were two herds of different mtDNA and nuclear genotypes to continuously inhabit adjacent ranges for hundreds of generations, the two locales would become indistinguishable in terms of nuclear markers (even at the fastest-evolving nuclear markers) due to male-mediated gene flow. Yet since females are not typically exchanged between two herds, in principle there might be no mitochondrial gene flow between the locales, and even between adjacent herds the mtDNA genetic differences would persist and might even increase due to novel mutations within each lineage. The combination of matrilocal social structure and intense male reproductive competition greatly increases the effective population size and coalescent of the mtDNA genome relative to nuclear loci [54], [55]. Thus mtDNA could provide a signal of ancient population partitioning that would not be at all reflective of the overall current population structure [55].

By contrast, not a single nuclear locus is “bound to the herd” like mtDNA. Males in one generation can transmit nuclear alleles from their natal herd to other herds. This occurs regardless of whether or not males traverse long distances. Males of one generation may transmit nuclear alleles to a nearby herd; their male offspring can then transmit them to even more distant herds, and thus nuclear alleles have the potential to traverse the African landscape. One notable point in this regard is that females do transmit their nuclear genetic endowment to male offspring, thus all of their nuclear alleles (autosomal or X-linked) thereby become easily transmitted across the landscape. Among genetic markers, only the mtDNA molecule is subject to the severe demographic constraint of being bound to the herd. All other elephant genetic markers are geographically unbound and subject to widespread and (compared to mtDNA) relatively rapid transmission across the geographic landscape [54]. Since the mtDNA would be expected to have a phylogeographic pattern that is unique and different from that of any other genetic loci, use of this marker in elephants should have been expected to lead to quite faulty inferences regarding their overall population structure or systematics [55]. Similar incongruence between mtDNA and nuclear phylogeographic patterns has been reported among a number of other mammalian taxa and, in cases where two species hybridize, genetic markers transmitted by the more highly dispersing sex were found to better delimit species [56].

### Reconciling the phylogeography of mtDNA and nuclear DNA in African elephants

We have quantified the relationship between the proportion of elephants in a population that carry S clade savanna mtDNA and the proportion of the population assigned to forest- or savanna-elephant nuclear genetic partitions (Figure 5). Surprisingly, the presence of S clade mtDNA among even a few elephants, even at low frequencies, or even in a nearby location (Figures 4 and 5), was sufficient to confer overwhelmingly savanna elephant nuclear genotypes to the elephants (Figure 5). By establishing the identity or similarity of mtDNA designations that differed across studies (Figure 3 and Figure S2), we could also analyze the geographic distribution of F clade and S clade mtDNA for hundreds of previously sequenced haplotypes in 81 locations across the ranges of forest and savanna elephants. This revealed that S clade mtDNA was only carried by elephants across the traditionally accepted range of the savanna elephant. S clade mtDNA was completely absent among elephants across the traditional range of the forest elephant. Previous discrepancies between mtDNA patterns and those of nuclear loci or morphology could thus begin to be resolved by considering the distribution of mtDNA haplogroups in populations rather than individuals [12]. The pattern we found lends support to a hypothesis that the source of nuclear alleles present in savanna but not forest elephants was the population in which the mtDNA S clade evolved [1], [4], [5], [25].

### The integrity of two species was maintained despite a historically shifting hybrid zone

Some previous studies have made the (often unstated) assumption that the presence of both F (forest elephant-derived) and S (savanna elephant-derived) mitochondrial DNA clades at a locale is an indication that the population of elephants at that locale must comprise a mixture of forest and savanna elephant genotypes [12], [16]. By contrast, we find that the mtDNA is an incongruent locus, not representative of the overall genetic makeup of the populations (Figure 5). In savanna locales where F clade mtDNA haplotypes are carried by a majority of the elephants the savanna elephant populations nonetheless have little or no discernible forest elephant contribution to nuclear genotypes (Figure 5). Other than carrying F clade mtDNA, these elephants proved not to be hybrids (Figures 1, 2) [57], with most savanna individuals carrying F clade mtDNA nonetheless displaying *ca.* 100% assignment of their genotypes into the savanna elephant partition in STRUCTURE analyses (Figures 2, 5). Even in Garamba, which demonstrates the greatest degree of hybridization among our sampled locales, the majority of individuals partitioned completely as forest elephants (Figure 2). This might be consistent with hybridization being a response to recent destruction of forests in Garamba [34], which may have permitted migration of savanna elephants into previously forested habitat and recent nuclear introgression [58]. Even in Garamba, a panmictic blend of forest and savanna elephants, in which all individuals would display a mix of forest and savanna genotypes, is not evident (Figure 2). One previous hypothesis, based on interpretations of mtDNA patterns, that “the hybrid zone between *africana* and *cyclotis* is not fairly ‘narrow’,” [12] is not supported by the current data, especially given the overlap of sampling locations (Cameroon, Zimbabwe) between the current and previous studies.

Isolation from savanna elephant gene flow appears to be complete among forest elephants, since in the current survey not a single forest elephant was found to carry S clade mtDNA across 30 forest locales in West or Central Africa. Previous studies had failed to detect, among a smaller survey of forest elephants, even a single Y-chromosome or X-chromosome haplotype derived from savanna elephants [4]. Although F clade mtDNA has crossed the species barrier and is often present in savanna elephants (Figures 3, 4, 5), the reverse pattern has not occurred to any detectable degree in our numerically large and geographically extensive current reanalysis of previously sequenced [4], [12], [13], [15], [16], [36], [37] forest elephants. Given the presence of hybrid individuals where the two types of elephant currently meet, and given the extensive historic hybrid zone suggested by the geographic expanse of F clade mtDNA [1], [4], [12], the complete absence of any detectable savanna elephant nuclear or mtDNA haplotype introgression into elephant populations in the tropical forests strongly suggests that isolating mechanisms between species [1], [25], [46] have prevented savanna or hybrid elephants from successfully contributing to the forest elephant gene pool.

The situation is more complicated among savanna elephants. Forest elephant derived F clade mtDNA has a very broad geographic range that extends long distances away from current forest habitats. F clade mtDNA is carried by *ca.* 20% of savanna elephants [4], [12], [13], as far north as Mali, east into Tanzania, and is carried by a majority of elephants in some locations as far south as Botswana and Zimbabwe [4], [12], [13], [15], [16]. Given the lack of any nuclear genetic evidence for hybridization in these regions (Figure 5), the presence of F clade mtDNA in these locales is likely an indication that the hybrid zone separating forest and savanna elephants has shifted [58] as climate and the distribution of forest habitats shifted during the geological history of Africa [4], [59], [60], [61], [62]. While at any point in time the hybrid zone may have remained narrow even as habitats shifted, nonetheless the very large geographic distribution of F clade [1], [4], [12] suggests that the hybrid zone shifted across a vast expanse of the African continent, suggesting that many generations of hybridization generated the current mtDNA pattern. Nonetheless, the genetic integrity of forest and savanna elephants has remained intact (Figure 5). This would also be consistent with the presence of species isolation mechanisms restricting nuclear gene flow between forest and savanna elephants [1], [25], [46]. Maintenance of genetic integrity and isolation in amidst such a hybrid zone would define species even under the biological species concept [46], [47], [48], as has been noted previously [1].

The historical reproductive success of female hybrids can be inferred by the presence of F clade mtDNA, derived from forest elephants, in these savanna elephant herds (Figures 3, 5) [1], [4], [12], [13], [25], [33]; while the failure of male hybrids to reproduce was inferred from the complete or nearly complete lack of forest elephant contribution to the nuclear genetic makeup of the savanna populations carrying forest-derived mtDNA (Figure 5) [1], [4], [13], [33]. The mito-nuclear pattern observed in savanna elephants can only result if male forest-savanna elephant hybrids are (relatively) reproductively unsuccessful while hybrid females repeatedly backcross to savanna males [1], [4], [25]. These inferences would be consistent with Haldane's rule [63] *sensu lato*, a commonly observed phenomenon detected following hybridization between distant taxa, in which the deleterious effects of hybridization tend to have greater impact on the heterogametic sex (i.e. males in the case of mammals), since the mito-nuclear patterns suggest that hybrid females have been reproductively successful but hybrid males have not [4], [46], [63]. The inferred backcrossing of hybrid females to non-hybrid savanna males would over generations dilute out forest elephant typical nuclear alleles in the savanna population, hence removing the relationship between the apparent population structure revealed by examination of mtDNA patterns, and the true population structure that remains after a population is stripped of the presence of forest elephant-derived nuclear alleles [1], [4], [13]. The mtDNA patterns would be rendered misleading for inferring the overall population structure or taxonomic status of Africa's elephants (Figures 1, 2, 5) [4], [56], [57].

### The taxonomic status of West Africa's elephants

Although cautious in their interpretation [15], Eggert and colleagues have proposed the hypotheses that the elephants “of West Africa belong to a newly recognized and yet to be formally named species” [14], and that “West African populations are now genetically distinct from other forest and savanna elephants and have been on a different trajectory for more than 2 Myr” [15], based in part on the phylogeography of mtDNA control region haplotypes. Debruyne (2005) [36] has questioned this hypothesis, after finding West African elephants interspersed with Central African elephants on a phylogenetic reconstruction based on their mtDNA. It may also be relevant that markers that are 100% effective at assigning African savanna, Asian, and African forest elephant species, are nonetheless found to mis-assign many elephants between West and Central African locales (Figure 6). This suggested that genetic similarities were present between West African and Central African forest elephants, and fails to lend support to the hypothesis that West African elephants comprise a distinct species.

Eggert et al. (2002) [15] also had hypothesized that in West Africa the elephants “do not divide into separate forest and savanna forms”, while Johnson et al. (2007) [16] state that in West Africa “savannah elephants are indistinguishable at both the mitochondrial and morphological level from their forest counterparts.” These hypotheses are difficult to reconcile with the conclusion drawn by Groves (2000) based on morphometric measurements on elephant skulls from across Africa, that some West African elephants were “firmly confirmed as *L. cyclotis*” in discriminant analyses [64]. Photographic evidence has also suggested that both forest and savanna elephants can be distinguished in West Africa [64], [65]. Among the highly disrupted habitats of West Africa [17], [66], [67] forest elephants currently range into some regions outside the tropical forest [64], [65]. However, historical surveys have concluded that most elephants in West Africa were eradicated by the first decades of the twentieth century, due to the ivory trade [66], [67], [68]. The impact was greater on savanna than on forest populations [66], [67]; thus one cannot readily assume that current geographic distributions of the elephant species in West Africa reflect their historic patterns.

Eggert et al. (2002) [15] had inferred nuclear relationships among their elephants using four STR loci, with the highest bootstrap values on nuclear phylograms supporting a clade that grouped the West African elephants of Mali with those of nearby savanna populations in Waza and Bénoué in Cameroon. It may not be possible to draw strong inferences from just four STR loci (especially as the placement of some populations such as Addo had likely been affected by inbreeding). Nonetheless, three of four STR loci genotyped by Eggert et al. (2002) [15] show similar allele sizes between Mali and either Waza or Bénoué (or both). The fourth locus was not similar in allele sizes between Mali and the Cameroon locales; however for locus *LA5*, the frequency of allele 191 was 1.00 in Mali and 0.46 in Waza, but 0.00 in all other savanna or forest locales sampled by Eggert et al. (2002) [15]; for locus *LA6*, the three highest frequencies for allele 172 were found in Mali (1.00), Waza (0.50), and Bénoué (0.27); and for locus *LafMS02*, the three highest frequencies for allele 150 were also in Mali (1.00), Waza (0.50), and Bénoué (0.27). Since Waza and Bénoué elephants have been shown by all nuclear DNA analyses including the current ones to partition as savanna elephants (Figures 1, 2, 5, 6), this suggests that the West African elephants in Mali may be genetically similar to other savanna elephants, a suggestion supported by the presence of S clade mtDNA in Mali (Figure 4, arrow). Thus, in the published STR dataset [15] it is difficult to find strong evidence for the hypothesis that Mali elephants may belong to a different species than Cameroon savanna elephants.

### “Mitochondrial essentialism” and the conservation of Africa's elephants

Given that mtDNA haplotypes among elephants are an unreliable indicator of overall genetic similarity (Figure 5) [4], it is unfortunate that mtDNA alone continues to be used as a guide to elephant genealogical affinities. This “mitochondrial essentialism,” the continuing use of mtDNA to partition populations and species, among elephants where morphological and nuclear markers have established that mtDNA patterns may be inaccurate or misleading, might lead to adverse results for elephant conservation, as the following examples illustrate: If mtDNA data were used as the sole basis for elephant taxonomy and population structure, elephants in the Guinean forest block could be recommended for translocation to the deserts of Mali, on the grounds that their mtDNA similarity implies that they must be genetically similar. Likewise, relying on mtDNA to infer population structure would mean that savanna elephants from Tanzania could be moved west into the Congolian tropical forest, since forest and savanna elephants in these regions share similar F clade mtDNAs. Either of these translocations would be inappropriate, since even while carrying mtDNA from the same haplogroup, individuals in forest and savanna locations are very different in nuclear genotypes (Figure 5), belong to different species [4], and are thus unlikely to thrive when moved to the wrong habitats. Although the examples are extreme, it may be equally troublesome that mtDNA-based misinterpretations of African elephant taxonomy constitute an unacknowledged potential hindrance to their proper conservation by convincing conservation groups to “continue to treat African elephants as a single species” [17].

### Improving the utility of nuclear genetic markers for fighting the ivory trade

Along with the destruction of habitats, one of the major threats to elephant populations is illegal hunting for the ivory trade. DNA has been successfully extracted from ivory [22], [23], while previous studies have shown that STR genotypes can be used to assign ivory to its geographic region of origin [20], [21], [24], [26]. However, previous studies have not attempted to rate each STR locus for its utility in assignment. In an attempt to further improve STR-based assignment methods, our STR loci were subjected to a novel analysis to assess their Shannon Information Content (SIC) using pairwise population comparisons to quantify their ability to distinguish elephants living in one geographic region or location from those living in a second region or location, which would gauge their utility for ivory forensics. For each comparison, some loci had a much higher SIC than others (Figure 7), suggesting that some STR loci would be much better than others for establishing the provenance of ivory. Our method permits the quantification of previously developed and current STR loci, and should permit the assessment of novel markers before they are brought into widespread use. Those loci that demonstrate an enhanced ability to distinguish among geographic locales can be chosen for inclusion in STR panels for ivory forensics. Our analysis makes it possible to develop a panel of STR markers, each component of which has a much a higher SIC than the current (randomly chosen) markers, and thus more effective for inferring the provenance of illegally poached ivory. Increased accuracy of assignment would enhance the ability of conservation, anti-corruption and law-enforcement efforts to identify elephant populations being targeted by poachers. Thus enhanced STR panels have the potential of aiding the conservation of Africa's two species of elephant.

## Materials and Methods

### Samples

The study was conducted in compliance with the University of Illinois Institutional Animal Care and Use Committed (IACUC) approved protocol number 09036. Samples were obtained in full compliance with required CITES (Convention on International Trade in Endangered Species of Wild Fauna and Flora) and other permits. As previously described, samples were collected from wild African elephants (*Loxodonta*) primarily by biopsy darting [11], [27], [69] of individuals from distinct herds. Blood samples were collected from Asian elephants and from Coco, a male forest elephant (*Loxodonta cyclotis*) from Sierra Leone, which had been kept at the Paris Zoo (Parc Zoologique de Paris-Vincennes, France). DNA was extracted primarily using kits (Qiagen), or standard phenol-chloroform methods [70]; samples of dung were extracted with DNA using a QIAmp DNA Stool Mini Kit (Qiagen).

### Microsatellite loci

Fifteen microsatellite loci were amplified (Supplementary Table S1). Five loci were from published sources [71], [72]; six loci were tetra-nucleotide microsatellites isolated from a savanna elephant using a capture hybridization method as previously described [73], with their sequences deposited in GenBank (accession numbers JF692777–JF692782); four loci with STRs were identified in savanna elephant DNA sequences mined from the NIH Comparative Vertebrate Sequencing Project (http://www.nisc.nih.gov/open_page.cgi?path=/projects/comp_seq.html). Microsatellites were identified using a repeat finder script written in Perl. PCR primers for novel STR loci were designed using Primer3 (http://fokker.wi.mit.edu/primer3/input.htm). PCR primers were tagged for fluorescence detection [74]. PCR amplification used a touchdown protocol as previously described [75]. Samples were genotyped on an ABI 3100 Genetic Analyzer and analyzed with Genescan 3.7 and Genotyper 2.5 programs (Applied Biosystems). Microsatellite data was binned in Allelogram [76] (http://code.google.com/p/allelogram).

A total of 15 loci were amplified in 555 African and 9 Asian elephants. The data was analyzed for deviation from Hardy–Weinberg equilibrium or excess of null alleles using Cervus 3.0 [77] (http://www.fieldgenetics.com/pages/aboutCervus_Overview.jsp). Two of the novel loci developed for this study from a capture hybridization library (LAF4 and LAF6, also designated p04 and p06) were excluded from some analyses because they exhibited an excess of homozygosity due to null alleles. Loci were examined for linkage using GENEPOP [78] (http://genepop.curtin.edu.au). Loci LAF30 and LAF35 (also designated p30 and p35, respectively) were excluded because of potential linkage to the more diverse loci LAF29 (p29) and LAF37 (p37), respectively.

### Microsatellite analyses

We used the program STRUCTURE 2.3.1 to apply a model-based clustering algorithm to identify subgroups of individual elephants that have distinctive allele frequencies [30], [79]. Each combination of species or locales was run 5 times using values of *K* between 1 and 21 genetic clusters, without any prior population information. Each analysis was run for at least 1 million Markov chain Monte Carlo generations following a burn-in of at least 100,000 steps. STRUCTURE was run using four models [30] combining assumptions on the genetic ancestry of individuals (i.e. the genome of a given individual is allowed –or not– to originate from more than one genetic cluster) and the genetic relatedness among populations (i.e. in the case of recent population subdivision allele frequencies in different populations are correlated): admixture–correlated, admixture–independent, no admixture–correlated, and no admixture–independent; with an inferred Dirichlet α parameter for population admixture (low α values suggest that most individuals do not have an admixed genome, while α>1 indicates that most individuals are of admixed genetic ancestry). Results were similar in each case. We estimated the uppermost hierarchical level of clusters using the *ad hoc* statistic Δ*K* based on the rate of change in the log probability of the data between successive values of *K* [31]. A multivariate representation of the analyzed individuals was carried out by subjecting data from 11 STR loci to Factorial Correspondence Analysis (FCA) in GENETIX 4.02 [35]. Genetic relationships among sampling locales were estimated using the chord distance [80] calculated in GenoDive [81], and visualized on a neighbor-joining tree [82] calculated in PAUP* 4b10 [83].

### Mitochondrial DNA sequencing and analyses

PCR amplifications using DNA from Bili Forest (BF) elephants were performed using 0.4 uM final concentration of each primer, 1.5 mM MgCl_2_, 200 uM of each dNTP (Applied Biosystems Inc. [ABI]) and 1 ug/ul final concentration of bovine serum albumin (BSA; New England BioLabs Inc.) with 0.04 units/ul final concentration of AmpliTaq Gold DNA Polymerase (ABI). PCR consisted of an initial denaturation at 95°C for 9∶45 min; cycles of denaturation for 20 sec at 94°C, annealing for 30 sec at 60° (initial 3 cycles), 58°C, 56°C, 54°C, 52°C (5 additional cycles each temperature), or 50°C (last 22 cycles), and 1 min extension at 72°C; with a final extension of 7 min at 72°C. PCR amplicons were examined on a 1% agarose gel and were enzyme-purified and sequenced using the BigDye Terminator v3.1 Cycle Sequencing Kit (ABI). Eight BF samples were successfully amplified and sequenced with amplicon sizes of 726 bp (681 bp excluding primers) or 375 bp (322 bp excluding primers), using the following primers: for the 726 bp amplicon, PCR primers were CBCR-F5 ATTACAATGGTCTTGTAAGCCATAAA and CBCR-R1d CTCAGACGGCCATAGCTGA; sequencing used the PCR primers and CBCR-F6 GATAAACCATAGTCTTACATAGCACAT and CBCR-R5 CTTTAATGTGCTATGTAAGACTATGG. For the 375 bp amplicon, PCR and sequencing primers consisted of CBCR-F5 and -R5. Novel mtDNA sequences have been deposited in Genbank (accession numbers: JF827273–JF827275). NCBI Blast (http://www.ncbi.nlm.nih.gov/blast/Blast.cgi) and comparison to previously sequenced elephant mtDNA were used to identify all BF elephant mtDNA sequences as F clade (Supplementary Figure S2). Fisher's exact test (two-tailed; http://www.graphpad.com/quickcalcs/contingency1.cfm) was used to analyze a 2×2 contingency table for association between the presence or absence of S clade mtDNA and the type of habitat at a locale (tropical forest vs. non-tropical forest or mixed).

### Shannon Information Content

Shannon Information Content (SIC) measures the amount of information contained in a message [43]. The greater the information in a message, the less random noise it contains, so the information can be referred to as a reduction in uncertainty [45]. Information thus becomes a measure of the improbability of an event. Specifically, Shannon defined information in terms of the base-2 logarithm of the reduction in uncertainty [45]. In terms of genetic markers, a very improbable allele in a locus is therefore assigned very high information content, since the information is comprised of the uncertainty that is eliminated by the appearance of the allele. The SIC considers the influence of centrality, whereby alleles that are absent (or nearly so) in one population are more informative than those common in both populations.

SIC has been successfully adapted to genetic studies, particularly for identifying genetic markers with frequencies highly specific for human populations of different geographic origins; which are used in admixture mapping of the human genome [42], a proven approach for mapping genetic variants that are involved in human disease [84]. In order to quantify the ability of different genetic markers to distinguish between elephants from different species and different regions or locales, we applied a routine described in Smith and O'Brien (2005) [42] to determine the Shannon Information Content. This was implemented in the statistical package SAS 9.1 (SAS Inc., Cary, NC), while Microsoft Excel was used to generate figures for comparisons among all markers: Supplementary Table S4 includes the contingency table, formulas and description of the routine.

## Supporting Information

Figure S1 The groups identified by Johnson et al. (2007) [16] based on African elephant mtDNA *CYTB* sequences largely overlap the mtDNA diversity reported by Debruyne (2005) [12]. Panel descriptions are shown in the figure.
(PDF)

Figure S2 Comparison of mtDNA sequences across studies of African elephant genetics: (A) Diagram illustrating mtDNA regions sequenced by different researchers, showing overlapping and non-overlapping regions across studies. Positions follow those of the elephant reference mtDNA genome (Genbank accession number NC_000934) [85]. (B) Corresponding clade designations for representative elephant sequences across studies. Relevant sequences that proved identical or matched closely across studies are indicated. The *ND4-tRNAGLU* sequences of Lei et al. (2008) [13] were used in a Blast query to retrieve the overlapping *ND5* sequences Roca et al. (2005) [4]. The *CYTB* sequences of the same individual elephant (same “sample ID”) of Lei et al. (2008) [13] was also used to retrieve matching *CYTB* and control region (CR) sequences of Debruyne (2005) [12] and *CYTB* sequences of Johnson et al. (2007) [16]. Through a Blast query using the retrieved Debruyne (2005) [12] sequence, matching mtDNA hypervariable region (HVR) sequences of Johnson et al. (2007) [16] were also obtained. Using this system, designations for the equivalent clades across studies were identified.
(PDF)

Figure S3 Map and list of locations of elephants sampled and sequenced for mtDNA across genetic studies [4], [12], [13], [15], [16], [36], [37].
(PDF)

Table S1 Fifteen elephant short tandem repeat loci amplified [71], [72].
(PDF)

Table S2 Allele range, number and frequency for 11 STR loci.
(PDF)

Table S3 Calculation of the *ad hoc* Evanno et al. (2005) [31] method for examining the true number of population subdivisions.
(PDF)

Table S4 Contingency table, formulas and other information for the Shannon Information Content.
(PDF)
