# Supplementary material for: Reconciling Apparent Conflicts between Mitochondrial and Nuclear Phylogenies in African Elephants
Source: PLoS One. 2011 Jun 8;6(6):e20642. doi: 10.1371/journal.pone.0020642 (PMC3110795; doi:10.1371/journal.pone.0020642)
Supplement: Figure S1 — The groups identified by Johnson et al. (2007) [16] based on African elephant mtDNA CYTB sequences largely overlap the mtDNA diversity reported by Debruyne (2005) [12]. Panel descriptions are shown in the figure. (PDF) [file pone.0020642.s001.pdf]

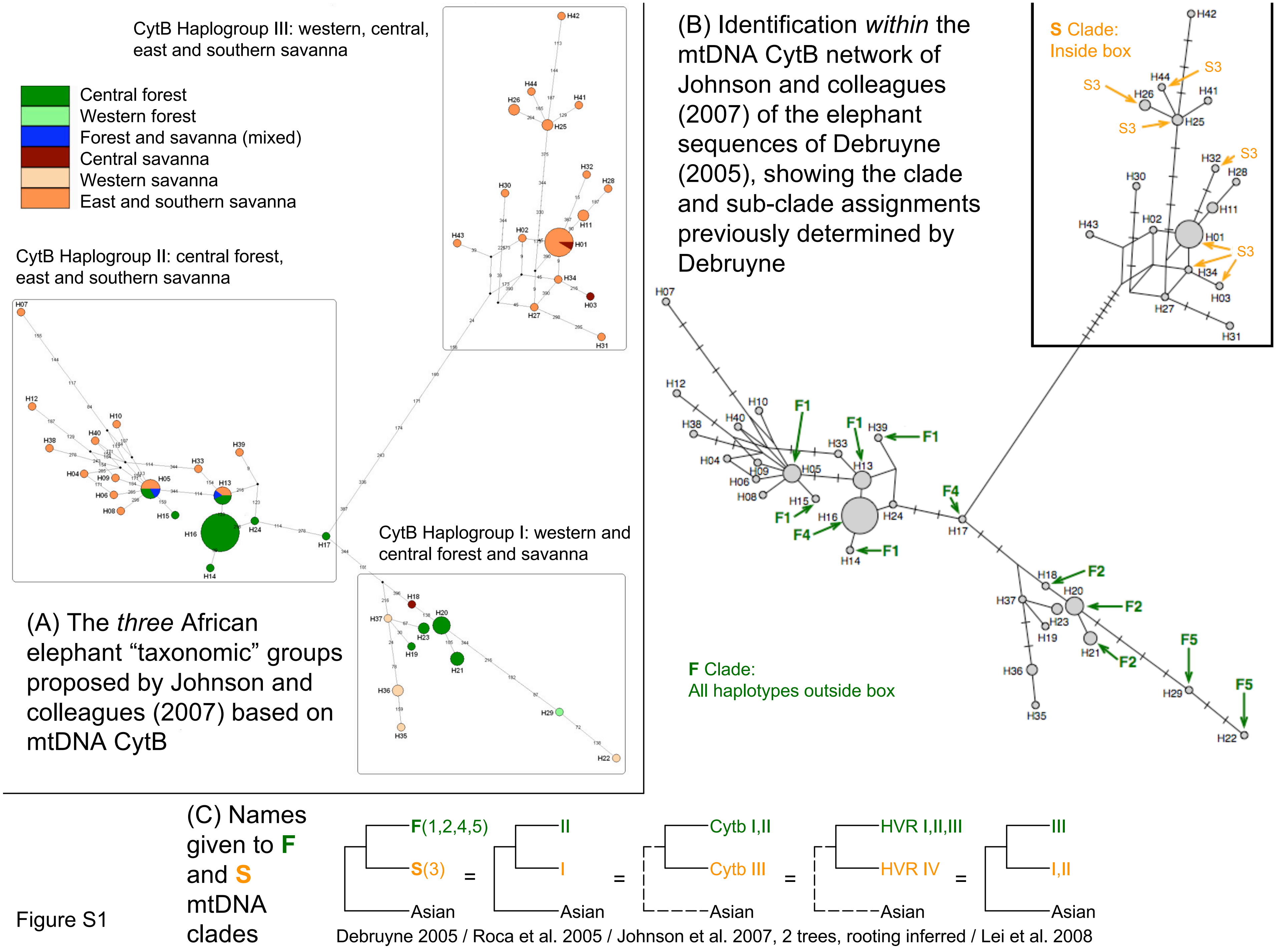
CytB Haplogroup III: western, central, east and southern savanna
Central forest
Western forest
Forest and savanna (mixed)
Central savanna
Western savanna
East and southern savanna
CytB Haplogroup II: central forest, east and southern savanna
CytB Haplogroup I: western and central forest and savanna
(A) The *three* African elephant "taxonomic" groups proposed by Johnson and colleagues (2007) based on mtDNA CytB
(B) Identification *within* the mtDNA CytB network of Johnson and colleagues (2007) of the elephant sequences of Debruyne (2005), showing the clade and sub-clade assignments previously determined by Debruyne
S Clade: Inside box
F Clade: All haplotypes outside box
(C) Names given to F and S mtDNA clades
F(1,2,4,5)
S(3)
Asian
II
I
Cytb I,II
Cytb III
HVR I,II,III
HVR IV
III
I,II
Debruyne 2005 / Roca et al. 2005 / Johnson et al. 2007, 2 trees, rooting inferred / Lei et al. 2008

Figure S1
