# Supplementary material for: Reconciling Apparent Conflicts between Mitochondrial and Nuclear Phylogenies in African Elephants
Source: PLoS One. 2011 Jun 8;6(6):e20642. doi: 10.1371/journal.pone.0020642 (PMC3110795; doi:10.1371/journal.pone.0020642)
Supplement: Figure S2 — Comparison of mtDNA sequences across studies of African elephant genetics: (A) Diagram illustrating mtDNA regions sequenced by different researchers, showing overlapping and non-overlapping regions across studies. Positions follow those of the elephant reference mtDNA genome (Genbank accession number NC_000934) [85]. (B) Corresponding clade designations for representative elephant sequences across studies. Relevant sequences that proved identical or matched closely across studies are indicated. The ND4-tRNAGLU sequences of Lei et al. (2008) [13] were used in a Blast query to retrieve the overlapping ND5 sequences Roca et al. (2005) [4]. The CYTB sequences of the same individual elephant (same “sample ID”) of Lei et al. (2008) [13] was also used to retrieve matching CYTB and control region (CR) sequences of Debruyne (2005) [12] and CYTB sequences of Johnson et al. (2007) [16]. Through a Blast query using the retrieved Debruyne (2005) [12] sequence, matching mtDNA hypervariable region (HVR) sequences of Johnson et al. (2007) [16] were also obtained. Using this system, designations for the equivalent clades across studies were identified. (PDF) [file pone.0020642.s002.pdf]

Figure S2 (A)

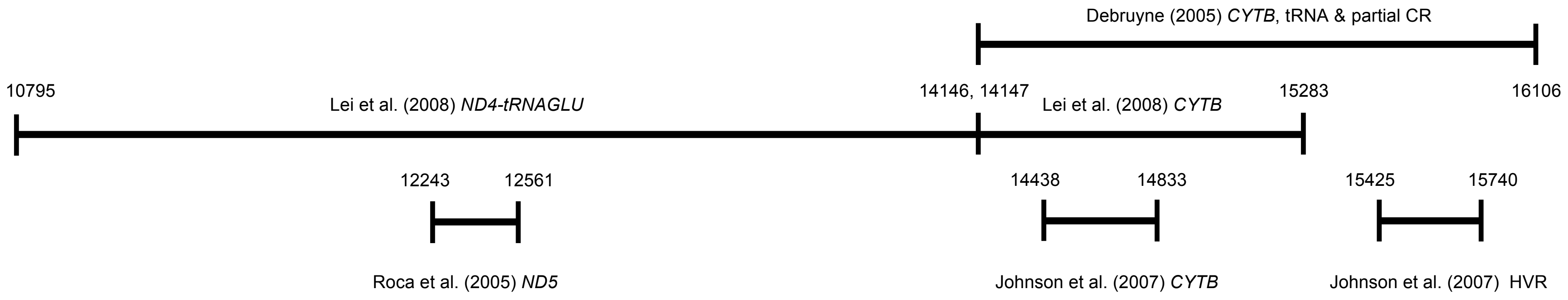
Debruyne (2005) *CYTB*, tRNA & partial CR
10795
14146, 14147
15283
16106
Lei et al. (2008) *ND4-tRNAGLU*
Lei et al. (2008) *CYTB*
12243
12561
14438
14833
15425
15740
Roca et al. (2005) *ND5*
Johnson et al. (2007) *CYTB*
Johnson et al. (2007) HVR

Figure S2 (B)

S Clade designations

mtDNA region	Debruyne (2005) *CYTB, tRNA & CR*	Debruyne (2005) Clade (Haplotype)	Roca et al. (2005) *ND5*	Roca et al. (2005) Clade	Johnson et al. (2007) *CYTB + HVR*	Johnson et al. (2007) Group (Haplotype)	Lei et al. (2008) *ND5/6 + CYTB*	Lei et al. (2008) Clade (Haplotype)	Lei et al. (2008) Sample ID
ND5			AY823346	**I**			AY781552	Savanna **II** (LAS1)	14
CYTB					EU116002 (99%)	**III** (H11)	AY768831	Savanna **II** (LAS1)	14
CYTB + CR	AY742801	**S** (Laa Tanzania1)			-	-			
ND5			AY823349	**I**			AY781638	Savanna **II** (LAS1)	365
CYTB					EU116004 (99%)	**III** (H27)	AY768917	Savanna **II** (LAS1)	365
CYTB + CR	AY741325	**S** (Laa Namibia1)			-	-			
ND5			AY823348	**I**			AY781562	Savanna **I** (LAS2)	62
CYTB					EU116014 (99%)	**III** (H41)	AY768841	Savanna **I** (LAS2)	62
CYTB + CR	AY741070	**S** (Laa Tanzania2)			-	-			
ND5			AY823348	**I**			AY781588	Savanna **I** (LAS2)	157
CYTB					EU116015 (99%)	**III** (H42)	AY768867	Savanna **I** (LAS2)	157
CYTB + CR	AY741322 (99%)	**S** (Laa Zimbabwe5)			-	-			

F Clade designations

mtDNA region	Debruyne (2005) *CYTB, tRNA & CR*	Debruyne (2005) Clade (Haplotype)	Roca et al. (2005) *ND5*	Roca et al. (2005) Clade	Johnson et al. (2007) *CYTB + HVR*	Johnson et al. (2007) Group (Haplotype)	Lei et al. (2008) *ND5/6 + CYTB*	Lei et al. (2008) Clade (Haplotype)	Lei et al. (2008) Sample ID
ND5			AY823341	**II**			AY781619	Forest **III** (LAF6)	218
CYTB					EU116000 (99%)	**II** (H09)	AY768898	Forest **III** (LAF6)	218
CYTB + CR	AY742802	**F** (DRC17)			EU096126 (97%)	**I** (H20)			
ND5			AY823342	**II**			AY781599	Forest **III** (LAF5)	182
CYTB					EU116000	**II** (H09)	AY768878	Forest **III** (LAF5)	182
CYTB + CR	AY741329 (99%)	**F** (Laa Zimbabwe2)			EU096126 (97%)	**I** (H20)			
ND5			AY823342	**II**			AY781602	Forest **III** (LAF2)	189
CYTB					EU116011 (99%)	**I** (H37)	AY768881	Forest **III** (LAF2)	189
CYTB + CR	AY359277(99%)	**F** (Lac DRC1)			EU096122 (98%)	**II** (H33)			

Note that sequences comprising Groups III and IV of the HVR from Johnson et al. (2007) were from Nyakaana et al. (2002), Eggert et al. (2002), Debruyne et al. (2003) and Debruyne (2005) (indicated as a dashes in the table). Note also that the clade designations in the Appendix of Lei et al. (2008) for haplogroups Savanna I and II appear to be reversed from the designations in the main text (the main text designations are used here). For sequence comparisons that did not show 100% match, the percent identity is shown in parentheses.
