# Supplementary material for: Reconciling Apparent Conflicts between Mitochondrial and Nuclear Phylogenies in African Elephants
Source: PLoS One. 2011 Jun 8;6(6):e20642. doi: 10.1371/journal.pone.0020642 (PMC3110795; doi:10.1371/journal.pone.0020642)
Supplement: Figure S3 — Map and list of locations of elephants sampled and sequenced for mtDNA across genetic studies [4], [12], [13], [15], [16], [36], [37]. (PDF) [file pone.0020642.s003.pdf]

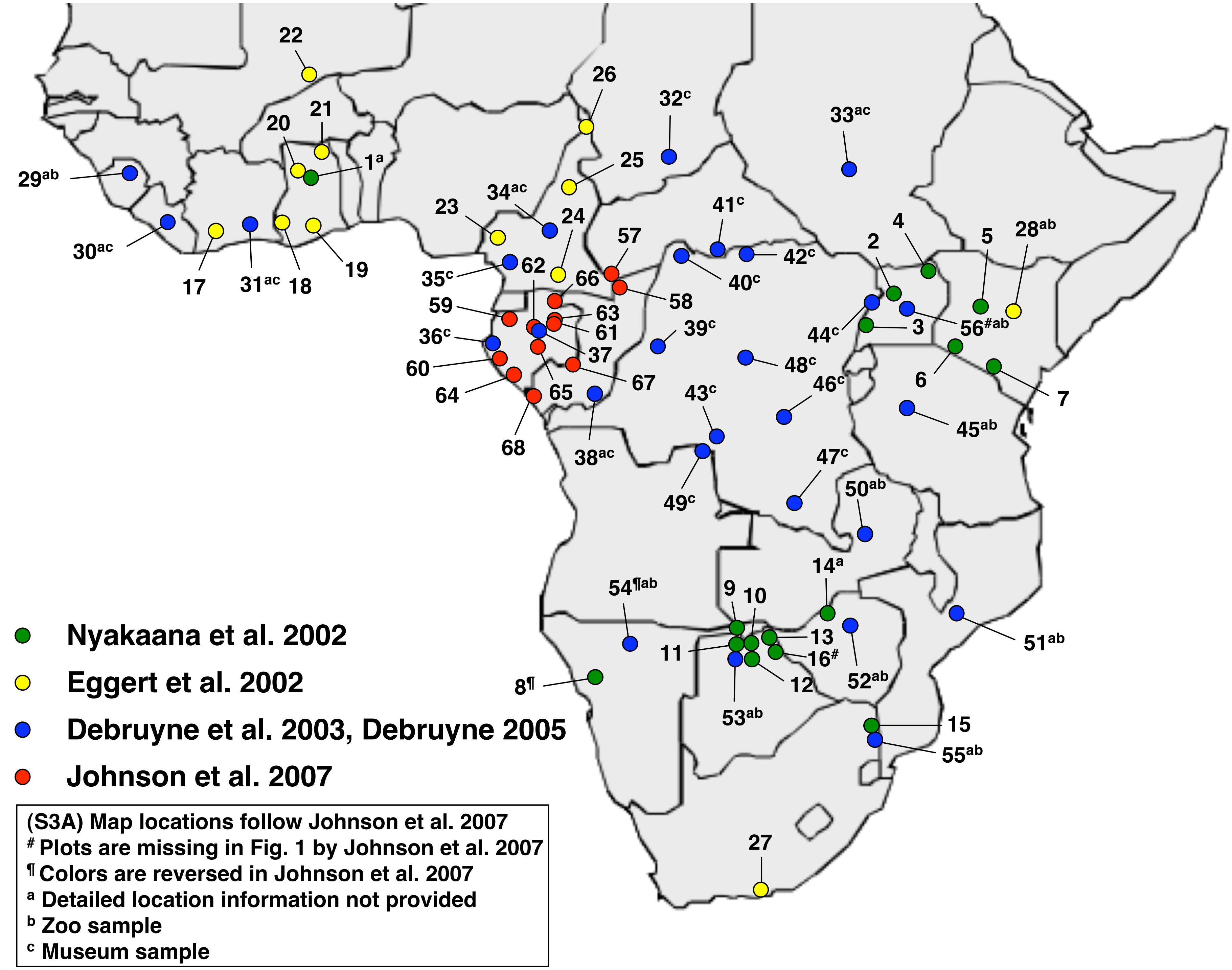

22
21
20
1[a]
29[ab]
30[ac]
17
31[ac]
18
19
26
25
32[c]
33[ac]
34[ac]
23
24
41[c]
57
35[c]
62
66
58
40[c]
42[c]
4
2
5
28[ab]
59
63
61
39[c]
44[c]
3
56[#ab]
36[c]
37
60
64
65
67
48[c]
6
7
46[c]
43[c]
45[ab]
68
38[ac]
49[c]
47[c]
50[ab]
14[a]
54[¶ab]
9
10
13
11
16[#]
12
52[ab]
51[ab]
8[¶]
53[ab]
15
55[ab]
27
Nyakaana et al. 2002
Eggert et al. 2002
Debruyne et al. 2003, Debruyne 2005
Johnson et al. 2007
(S3A) Map locations follow Johnson et al. 2007
Plots are missing in Fig. 1 by Johnson et al. 2007
¶ Colors are reversed in Johnson et al. 2007
a Detailed location information not provided
b Zoo sample
c Museum sample

Figure S3 (B): List of sample locations on map.

No.	Country	Location	Sample origin
Nyakaana et al. 2002			
1	Ghana		Wild
2	Uganda	Murchison Falls NP	Wild
3	Uganda	Queen Elizabeth NP	Wild
4	Uganda	Kidepo Valley NP	Wild
5	Kenya	Samburu GR	Wild
6	Kenya	Masai Mara NR	Wild
7	Kenya	Amboseli NP	Wild
8*¶	Namibia	Khorixas	Wild
9*	Namibia	Caprivi NP	Wild
10*	Botswana	Chobe NP	Wild
11*	Botswana	Kwando	Wild
12*	Botswana	Nunga Valley	Wild
13*	Botswana	Sibuyu FR	Wild
14*	Zimbabwe		Wild
15*	South Africa	Kruger NP	Wild
16*#	Botswana	Ngwasha	Wild
Eggert et al. 2002			
17	Côte d'Ivoire	Tai NP	Wild
18	Ghana	Bia NP	Wild
19	Ghana	Kakum NP	Wild
20	Ghana	Mole NP	Wild
21	Ghana	Red Volta Valley	Wild
22	Mali	Gourma Region	Wild
23	Cameroon	Banyang Mbo Wildlife Sanctuary	Wild
24	Cameroon	Dja FR	Wild
25	Cameroon	Benoue NP	Wild
26	Cameroon	Waza NP	Wild
27	South Africa	Addo Elephant NP	Wild
28	Kenya		Zoo
Debruyne et al. 2003, Debruyne 2005			
29ˆ	Sierra Leone		Zoo
30	Liberia		Museum
31	Côte d'Ivoire		Museum
32	Chad	Zakouma NP	Museum
33	Sudan (ex-southern Egypt)		Museum
34	Cameroon		Museum
35	Cameroon	Yambong	Museum
36	Gabon	Coast near Aloombé	Museum
37	Gabon	Lope National Park	Wild
38	RC		Museum
39	DRC	Mai-Ndome Lake	Museum
40	DRC	Bosobolo	Museum
41	CAR	Ubangi River	Museum
42	DRC	Uele n' Dungu	Museum
43	DRC	Kamemba	Museum
44	DRC	Kanyatsi	Museum
45	Tanzania		Zoo
46	DRC	Panga Na Bodio	Museum
47	DRC	Katanga	Museum
48	DRC	Moma	Museum
49	Angola	Luiza (DRC frontier)	Museum
50	Zambia		Zoo
51	Mozambique		Zoo
52	Zimbabwe		Zoo
53	Botswana		Zoo
54¶	Namibia		Zoo
55	South Africa		Zoo
56#	Uganda		Zoo
Johnson et al. 2007†			
57	CAR	Dzanga-Sangha NP	Wild
58	RC	Noubalé-Ndoki NP	Wild

59	Gabon	Monts de Cristal	Wild
60	Gabon	Iguéla NP	Wild
61	Gabon	Ivindo NP	Wild
62	Gabon	Lope NP	Wild
63	Gabon	Ipassa Reserve	Wild
64	Gabon	Loango/Mayumba NP	Wild
65	Gabon	Massif de Chaillu	Wild
66	Gabon	Minkébé NP	Wild
67	Gabon	Plateaux Batéké NP	Wild
68	Gabon	Conkouati-Douli NP	Wild

<u>Roca et al. 2005</u>

Locales 69-81 (not numbered on the map of Johnson and colleagues)

The F and S clades were identified for this dataset. Locations are identified in Figure 1 of the main text, and were not added to the map derived from Johnson and colleagues (2007), reproduced above. For locales also sampled by others, the results of Roca and colleagues (2005) are consistent in terms of presence or absence of S clade mtDNA. Locations not sampled by others are listed here:

Botswana	Mashatu	Wild
Botswana	Savuti	Wild
DRC	Bili Forest	Wild
DRC	Garamba	Wild
Kenya	Aberdares	Wild
Kenya	Mount Kenya	Wild
RC	Odzala	Wild
Tanzania	Ngorongoro	Wild
Tanzania	Serengeti	Wild
Tanzania	Tarangire	Wild
Zimbabwe	Hwange	Wild
Zimbabwe	Sengwa	Wild
Zimbabwe	Zambezi	Wild

<u>Lei et al., 2008</u>

The F and S clades were identified for this dataset. Lei and colleagues used elephants from North American zoos, all of which are savanna elephants. They often can be assigned to a country but not a more precise location, and are almost all from countries in which wild elephants have been heavily genotyped for mtDNA. No additional locales were added to the map based on this study. However, where Johnson and colleagues (2007) had placed an icon on the map for zoo elephants, results were updated with the the additional data of Lei and colleagues (2008). This led to one update, at "locale" 28, where the presence of S clade is recorded for zoo elephants from Kenya.

<u>Abbreviations</u>

CAR (Central Africal Republic)
DRC (Democratic Republic of the Congo)
RC (Republic of the Congo)
NP (National Park)
NR (National reserve)
FR (Forest reserve)

<u>Notes</u>

*: Detailed locations follow Eggert et al. 2002.
#: Plots are missing in Fig. 1 by Johnson et al. 2007.
¶: Locations 8 and 54 have colors reversed in Fig.1 of Johnson et al. 2007.
† Locations and countries were not clearly indicated by Johnson et al. 2007.
ˆ Original GenBank data is from Barriel et al. 1999.
