# Supplementary material for: Reconciling Apparent Conflicts between Mitochondrial and Nuclear Phylogenies in African Elephants
Source: PLoS One. 2011 Jun 8;6(6):e20642. doi: 10.1371/journal.pone.0020642 (PMC3110795; doi:10.1371/journal.pone.0020642)
Supplement: Table S1 — Fifteen elephant short tandem repeat loci amplified [71], [72]. (PDF) [file pone.0020642.s004.pdf]

Table S1. Fifteen elephant short tandem repeat loci amplified

STR locus	Designation	Species origin	Repeat Motif	Forward sequence	Reverse sequence	Microsatellite Source
LAF4*	p4	Savanna elephant	(CCTA)9..(CCTA)19	TCTGTCTGTCTGTCTACCTC	AGATAAATAGATAGATAGGTGAT	Tetra-nucleotide repeat library, this study
LAF6*	p6	Savanna elephant	(CCTA)6	CTGGCGATTTTGAGGATGAT	TGGGTAGGTAGGCAGATACGA	Tetra-nucleotide repeat library, this study
LAF13	p13	Savanna elephant	(TCTA)5	CACAGGTACAGAAGCCAGGA	GGAGCCAGACCCTACAAATG	Tetra-nucleotide repeat library, this study
LAF10	p10	Savanna elephant	(GGAT)5(GGAC)3	GTTTCATGGGCCAGATTTTC	TGCCTTCTTACCTGTCTATCCA	Tetra-nucleotide repeat library, this study
LAF11	p11	Savanna elephant	(TAGA)5	AGCTGAGCTCTTAACCACTGC	TTGTTTCCAAACTTTTGCATTCT	Tetra-nucleotide repeat library, this study
LAF12	p12	Savanna elephant	(TCTA)14	TCTATGTATCTATCATCTGTCTATCTG	CATTCGGAAAGTCCAACACC	Tetra-nucleotide repeat library, this study
LAF29	p29	Savanna elephant	(CA)14	GCAGCCATTAGGAAAAGCAC	CAGAGGGAGCTTCAGGTGAG	Mined from NIH Comparative Vertebrate Sequencing Project, this study
LAF30**	p30	Savanna elephant	(TTG)11	GACATCAGCAAACTGCAGGA	GCATACCACTGATCGGGAAG	Mined from NIH Comparative Vertebrate Sequencing Project, this study
LAF35**	p35	Savanna elephant	(CA)15	TGGAGAAGGGGAGAGTGTTG	CTCATGGGCATCTAGTGCAA	Mined from NIH Comparative Vertebrate Sequencing Project, this study
LAF37	p37	Savanna elephant	(TG)14	GCACCAAGAGTTGGAAAGAGTT	TGTTTTTAGTTTCTACTTTGGAAACAG	Mined from NIH Comparative Vertebrate Sequencing Project, this study
LaT05	p17	Savanna elephant	(CCAT)2 CCAC (CCAT)14 CAT (CCAT)17	CACCACCCATCCATCTGT	TGGCTTCTGTGAGTTCACC	Archie et al. 2003
LaT06	p18	Savanna elephant	(CCAT)13	AGCCAGGCACATTAAGTGT	TCTCCTAGAAAAGGTTACCACA	Archie et al. 2003
EMX3	p14	Asian elephant	[GGAA]3..(GAA)5..GGAA)4	CATGGTTAACTCATTGCTTGC	GTGTTCCCTCCCTCTCATCAT	Fernando et al. 2001
EMX4	p15	Asian elephant	(GGAA)3 A (GA)3 A (GGAA)3	GTTCGTGTCTCGGTGCTGTA	GTATGCTGATGGAAATGTCTA	Fernando et al. 2001
EMX5	p16	Asian elephant	(GGAA)3..(GGAAGGGA)4..(GGAA)3..(AGGG)3	AAATAGGAAAAGTCTGAGGTT	CCCCTGGATTTTCTTCACCTG	Fernando et al. 2001

*locus dropped due to excess of homozygosity (null alleles)

**locus dropped from final anlyses due to linkage to another locus

Archie et al. 2003: Archie E.A., Moss C.J. and Alberts S.C. (2003). Characterization of tetranucleotide microsatellite loci in the African savannah elephant (*Loxodonta africana*). Molecular Ecology Notes. 3: 244-246.

Fernando et al. 2001: Fernando P., Vidya T.N.C., Melnick D.J. (2001). Isolation and characterisation of tri- and tetranucleotide microsatellite loci in the Asian elephant, *Elephas maximus*. Mol Ecol Notes 1: 232–233.
