# Supplementary material for: Reconciling Apparent Conflicts between Mitochondrial and Nuclear Phylogenies in African Elephants
Source: PLoS One. 2011 Jun 8;6(6):e20642. doi: 10.1371/journal.pone.0020642 (PMC3110795; doi:10.1371/journal.pone.0020642)
Supplement: Table S2 — Allele range, number and frequency for 11 STR loci. (PDF) [file pone.0020642.s005.pdf]

Table S2. Allele range, number and frequency for 11 STR loci

Locus*	a.k.a.*	Allele Range			Number of Alleles			Heterozygosity		
		Forest	Savanna	Asian	Forest	Savanna	Asian	Forest	Savanna	Asian
LAF10	p10	158-166	158-166	158-162	3	3	2	0.421	0.382	0.233
LAF11	p11	243-251	243-251	247	3	3	1	0.174	0.23	0
LAF12	p12	166-238	154-226	214-278	13	18	9	0.862	0.872	0.879
LAF13	p13	226-230	222-334	315-350	14	12	8	0.894	0.513	0.875
LAF29	p29	197-223	201-221	201-203	12	11	2	0.827	0.698	0.5
LAF37	p37	185-237	185-227	213-227	14	13	5	0.804	0.677	0.683
LaT05	p17	277-521	265-505	397-489	39	22	11	0.968	0.909	0.933
LaT06	p18	315-419	291-405	263-323	22	24	6	0.936	0.715	0.617
EMX3	p14	261-264	252-270	252-267	4	4	2	0.456	0.14	0.533
EMX4	p15	353-377	361-397	387-397	5	5	2	0.299	0.503	0.233
EMX5	p16	265-269	269-285	265-285	2	2	4	0.406	0.004	0.592

Note: based on 11 STR loci amplified in 75 forest, 537 savanna and 8 Asian elephants
*Both the published locus name and the working designation are listed
