# Supplementary material for: Reconciling Apparent Conflicts between Mitochondrial and Nuclear Phylogenies in African Elephants
Source: PLoS One. 2011 Jun 8;6(6):e20642. doi: 10.1371/journal.pone.0020642 (PMC3110795; doi:10.1371/journal.pone.0020642)
Supplement: Table S3 — Calculation of the ad hoc Evanno et al. (2005) [31] method for examining the true number of population subdivisions. (PDF) [file pone.0020642.s006.pdf]

Table S3: Calculation of the *ad hoc* Evanno et al. method for examining the true number of population subdivisions

Parameters-burnin-iterations	K	Ln	delta Ln	delta' Ln	elt' Ln/SD	P(D)	Var[LnP(D)]	a1	Fst_1	Fst_2	Fst_3	Fst_4
admixture.correlated.100k.1M	1	-17192.1	0	0	0	82.1	-	0.0001	-	-	-	-
admixture.correlated.100k.1M	2	-14455.5	2743.3	2562.1	11.8891	209.9	0.028	0.284	0.0199	-	-	-
admixture.correlated.100k.1M	3	-14264.3	181.2	73	0.17052	410.9	0.0321	0.0427	0.3035	0.2561	-	-
admixture.correlated.100k.1M	4	-14154.3	108.2	74.3	0.12402	591.6	0.0349	0.229	0.2714	0.0691	0.2357	-
admixture.correlated.100k.1M	5	-14103	33.9	43.6	0.05619	755.7	0.037	0.086	0.2473	0.2137	0.212	0.2175
admixture.independent.100k.1M	1	-17186.3	0	0	0	76.4	-	-	-	-	-	-
admixture.independent.100k.1M	2	-14440.9	2743.3	2562.1	11.8891	202.8	0.0283	-	-	-	-	-
admixture.independent.100k.1M	3	-14270.8	181.2	73	0.17052	425.2	0.0333	-	-	-	-	-
admixture.independent.100k.1M	4	-14164.5	108.2	74.3	0.12402	605.3	0.0367	-	-	-	-	-
admixture.independent.100k.1M	5	-14093.6	33.9	43.6	0.05619	745.4	0.0395	-	-	-	-	-

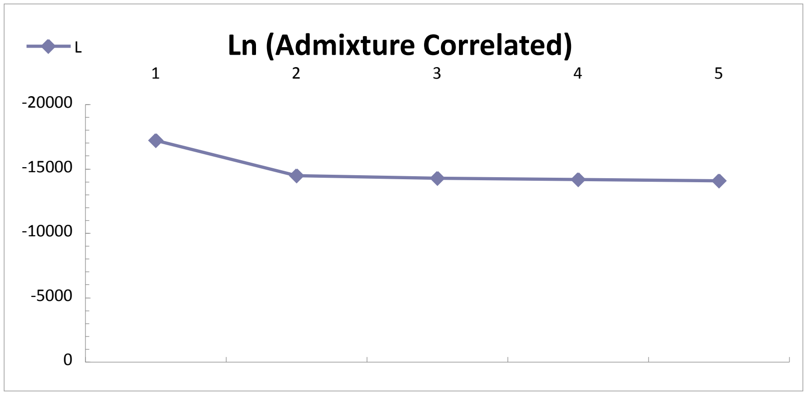

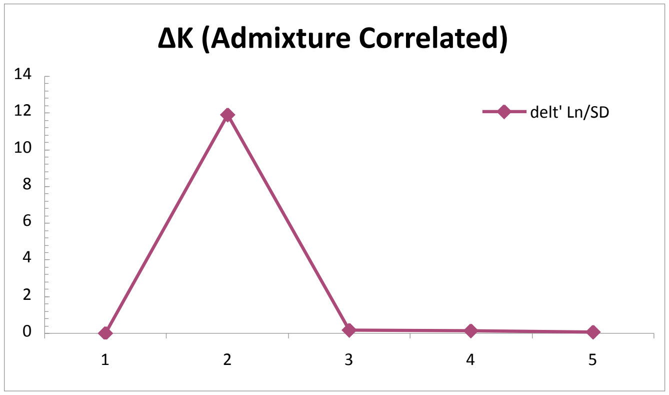

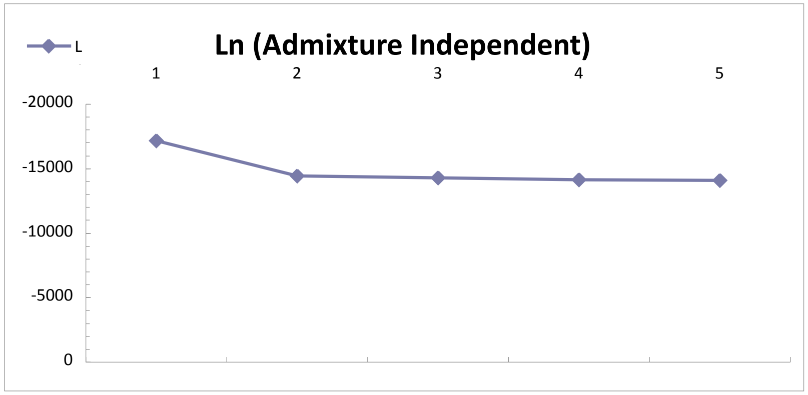

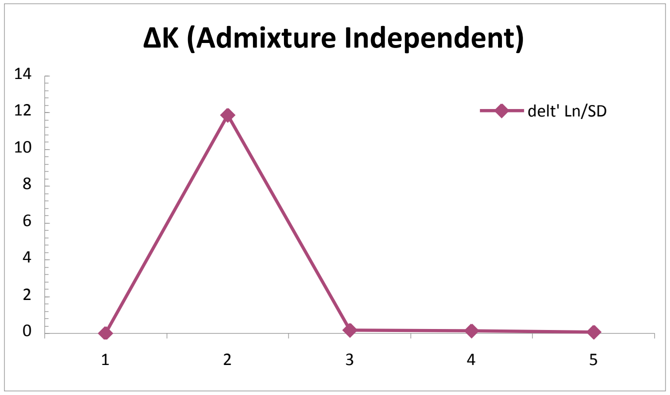

Reference: Evanno G, Regnaut S, Goudet J. 2005. Detecting the number of clusters of individuals using the software *STRUCTURE:* a simulation study. Mol Ecol. 14(8):2611-20
