# Supplementary material for: Reconciling Apparent Conflicts between Mitochondrial and Nuclear Phylogenies in African Elephants
Source: PLoS One. 2011 Jun 8;6(6):e20642. doi: 10.1371/journal.pone.0020642 (PMC3110795; doi:10.1371/journal.pone.0020642)
Supplement: Table S4 — Contingency table, formulas and other information for the Shannon Information Content. (PDF) [file pone.0020642.s007.pdf]

Supplementary Table S4. Contingency table, formulas and other information for the Shannon Information Content.

In order to quantify the ability of different genetic markers to distinguish between elephants from different species and different regions or locales, we applied a routine described in Smith and O'Brien (2005) [1] to determine the Shannon Information Content. This was implemented, based on the following formulas, in the statistical package SAS 9.1 (SAS Inc., Cary, NC), while Microsoft Excel was used to generate figures for comparisons among all markers:

	**First locale**	**Second locale**	**Total**
**Allele 1**	**$(1-m)f_A$** **$[a_{00}]$**	**mf_E** **$[a_{01}]$**	**$(1-m)f_A + mf_E$** **$[a_{0*}]$**
**Allele 2**	**$(1-m)(1-f_A)$** **$[a_{10}]$**	**$m(1- f_E)$** **$[a_{11}]$**	**$(1-m)(1-f_A) + m(1-f_E)$** **$[a_{1*}]$**
**Total**	**$1-m$** **$[a_{*0}]$**	**m** **$[a_{*1}]$**	**1**

$$SIC = \sum_{i=0}^{1}\sum_{j=0}^{1} a_{ij}\log_2 a_{ij} - a_{i*}\log_2 a_{i*} - a_{*i}\log_2 a_{*i}$$

For a case with only two locales this becomes:

$$SIC = -\sum_{i=0}^{1}(a_{i0} + a_{i1})\log(a_{i0} + a_{i1}) - \sum_{j=0}^{1}(a_{0j} + a_{1j})\log(a_{0j} + a_{1j}) + \sum_{i=0}^{1}\sum_{j=0}^{1} a_{ij}\log(a_{ij})$$

where $a_{00} = (1–m) \times p^{\mathrm{loc1}}$, $a_{01} = m \times p^{\mathrm{loc2}}$, $a_{10} = (1–m) \times (1–p^{\mathrm{loc1}})$, and $a_{11} = m \times (1–p^{\mathrm{loc2}})$. Here, p^{loc1} and p^{loc2} are the STR frequencies in the contrasted species, regions, populations or locales, and *m* is the proportion of loc2 “ancestry” in the “admixed” population, which was varied from 0 to 1. Alleles absent at a locale were coded as present in a single copy, to account for limited sampling.

When applied to studies of human diseases, the allelic frequencies for the populations of origin as well as the admixture proportion (*m*) between the two populations are often well known [2,3,4,5]. In contrast, for our comparisons of elephants from different geographic groups, we made no assumptions about “admixture” (*m*) between locales. Instead, the entire range of possible *m* proportions (from 0 to 1) was examined and plotted for each one of the STRs in the given species-to-species comparison. The best (maximum) value of SIC identified from this range was chosen and used to evaluate between STR loci against each other for their information content.

Previously, marker selection has been reported as not very sensitive to the choice of m [5].

References

1. Smith MW, O'Brien SJ (2005) Mapping by admixture linkage disequilibrium: advances, limitations and guidelines. Nat Rev Genet 6: 623-632.
2. Freedman ML, Haiman CA, Patterson N, McDonald GJ, Tandon A, et al. (2006) Admixture mapping identifies 8q24 as a prostate cancer risk locus in African-American men. Proc Natl Acad Sci U S A 103: 14068-14073.
3. Kopp JB, Smith MW, Nelson GW, Johnson RC, Freedman BI, et al. (2008) *MYH9* is a major-effect risk gene for focal segmental glomerulosclerosis. Nat Genet 40: 1175-1184.
4. Thorisson GA, Smith AV, Krishnan L, Stein LD (2005) The International HapMap Project Web site. Genome Res 15: 1592-1593.
5. Smith MW, Patterson N, Lautenberger JA, Truelove AL, McDonald GJ, et al. (2004) A high-density admixture map for disease gene discovery in African Americans. Am J Hum Genet 74: 1001-1013.
